# Diabetes-related information-seeking behaviour: a systematic review

**DOI:** 10.1186/s13643-017-0602-8

**Published:** 2017-10-24

**Authors:** Silke Kuske, Tim Schiereck, Sandra Grobosch, Andrea Paduch, Sigrid Droste, Sarah Halbach, Andrea Icks

**Affiliations:** 1 Institute for Health Services Research and Health Economics, Centre for Health and Society, Faculty of Medicine, Heinrich Heine University Düsseldorf, Moorenstraße 5, 40225 Düsseldorf, Germany; 2 Department for Psychosomatic Medicine and Psychotherapy, Center for Health Communication and Health Services Research (CHSR), University Hospital Bonn, Sigmund-Freud-Str. 25, 53127 Bonn, Germany; 3 Institute for Health Services Research and Health Economics, German Diabetes Center, Düsseldorf, Germany; 4 German Center for Diabetes Research, Neuherberg, Germany

**Keywords:** Diabetes, Information-seeking behaviour, Systematic review

## Abstract

**Background:**

Information-seeking behaviour is necessary to improve knowledge on diabetes therapy and complications. Combined with other self-management skills and autonomous handling of the disease, it is essential for achieving treatment targets. However, a systematic review addressing this topic is lacking. The aims of this systematic review were to identify and analyse existing knowledge of information-seeking behaviour: (1) types information-seeking behaviour, (2) information sources, (3) the content of searched information, and (4) associated variables that may affect information-seeking behaviour.

**Methods:**

The systematic review follows the Preferred Reporting Items for Systematic Reviews and Meta-Analyses (PRISMA) requirements. MEDLINE, CINAHL, EMBASE, ScienceDirect, PsycInfo, Cochrane Library, Web of Science, CCMed, ERIC, Journals@OVID, *Deutsches Ärzteblatt* and *Karlsruher virtueller Katalog* (KvK) databases were searched. Publications dealing with information-seeking behaviour of people with diabetes mellitus published up to June 2015 were included. A forward citation tracking was performed in September 2016 and June 2017. Additionally, an update of the two main databases (MEDLINE, CINAHL) was conducted, considering studies published up to July 2017. Studies published in languages other than English or German were excluded, as well as letters, short reports, editorials, comments and discussion papers. A study selection and the critical appraisal of the selected studies were performed independently by two reviewers. A third reviewer was consulted if any disagreement was found. Data extraction and content analysis were performed using selected dimensions of Wilson’s ‘model of information behaviour’.

**Results:**

Twenty-six studies were included. Five ‘types of information-seeking behaviour’ were identified, e.g. passive and active search. The ‘Internet’ and ‘healthcare professionals’ were the most frequently reported sources. ‘Diet’, ‘complications’, ‘exercise’ and ‘medications and pharmacological interactions’ were the most frequently identified content of information. Seven main categories including associated variables were identified, e.g. ‘socioeconomic’, ‘duration of DM’, and ‘lifestyle’.

**Conclusion:**

The systematic review provides a valuable overview of available knowledge on the information-seeking behaviour of people with diabetes mellitus, although there are only a few studies. There was a high heterogeneity regarding the research question, design, methods and participants. Although the Internet is often used to seek information, health professionals still play an important role in supporting their patients’ information-seeking behaviour. Specific needs of people with diabetes must be taken into consideration.

**Systematic review registration:**

PROSPERO CRD42016037312

**Electronic supplementary material:**

The online version of this article (10.1186/s13643-017-0602-8) contains supplementary material, which is available to authorized users.

## Background

In 2013, it was estimated that 382 million adults (18–79 years) worldwide had diabetes mellitus (DM), and this number is expected to rise to 592 million in 2035 [1]. DM is associated with high healthcare costs and comorbidities that can result in individual restrictions and in a reduced quality of life [2–4]. In this regard, the International Diabetes Federation estimated the costs of DM treatment in Europe at US$290 billion in 2015 [4].

Self-management is essential in achieving treatment targets, as is indicated by the outcomes of clinical studies regarding the percentage of glycated haemoglobin (HbA_1c_%) or blood pressure control [5]. An important aspect of self-management is patients’ knowledge, including their ability to seek information [6]. Information-seeking behaviour is one component of information behaviour along with handling sources and channels and using information. Wilson’s ‘model of information behaviour’ describes information seeking and use as direct consequences of information need [7], and characterises ‘types of information seeking’ and its related ‘intervening variables’ and considers ‘activating mechanism’ based on several psychological and social theories [8]. Within this context, this review focuses on information-seeking behaviour that is defined as ‘the purposive seeking for information as a consequence of a need to satisfy some goal’ [7]. According to Wilson’s model, there are four different types of information-seeking behaviour: passive attention, passive or active searching and ongoing search [8]. Passive attention is obtaining information without intending to look for it (e.g. watching television). Passive searching is finding relevant information while searching for other topics of information. This usually leads to active searching, ‘the principal mode’ in the process of information seeking, where ‘an individual actively seeks out information’ [8]. The last mode is ‘ongoing search’, which is performed during active search to update or to expand present information [8]. The intervening variables that are associated with information-seeking behaviour are ‘psychological’, ‘demographic’, ‘role-related or interpersonal’, ‘environmental’ variables and ‘source characteristics’ (e.g. currency, appropriateness) [8].

Research into information-seeking behaviour among people with DM appears to be limited so far, despite the fact that studies have demonstrated that information-seeking behaviour is crucial in enabling people to cope with the consequences of their disease [5]. Although there are some publications concerning information-seeking behaviour among individuals with DM [5, 9, 10], to the best of our knowledge, no systematic review has yet analysed knowledge on information-seeking behaviour. An overview of the information-seeking behaviour of people with DM is needed to develop recommendations for practice and research in order to guarantee the necessary support for handling information on diabetes.

The aims of this systematic review were to identify and analyse existing knowledge of information-seeking behaviour: (1) types of information-seeking behaviour, (2) information sources, (3) the content of searched information, and (4) associated variables, which may affect the information-seeking behaviour of people with all types of DM.

## Methods

A systematic review was performed in line with the Preferred Reporting Items for Systematic Reviews and Meta-Analyses (PRISMA) quality requirements [11] and is registered at the International Prospective Register of Systematic Reviews (PROSPERO) (CRD42016037312). A systematic review protocol was developed that guided the review process (Additional file 1).

### Search strategy

The systematic literature search was performed in MEDLINE, CINAHL, EMBASE, ScienceDirect, PsycINFO, Cochrane Library, Web of Science, CCMed, ERIC, Journals@OVID, DARE, ISI and EED. German sources were also searched: *Deutsches Ärzteblatt* and *Karlsruher virtueller Katalog* (KvK). Studies published up to June 2015 were considered. Additionally, an update of the two core databases (MEDLINE, CINAHL) was conducted, including studies published up to July 2017. The search strategy was developed using database-specific controlled vocabularies and free-text terms (Additional file 2). The search terms included for DM were, among others, ‘diabetes mellitus’, ‘diabetes mellitus, Type 1/Type 2’, ‘diabetic’, ‘niddm’, ‘iddm’, ‘t2dm’ and ‘t1dm’. The search terms included for information-seeking behaviour were, among others, ‘information-seeking behaviour’, ‘information behaviour’ and ‘isb’. Duplicates of all databases were removed. A forward citation tracking was performed in September 2016 and June 2017 to identify further relevant studies in Google Scholar by searching citations of already identified core publications [5, 12, 13]. Google Scholar locates articles by using a matching algorithm and searching keywords in title, abstract or full text [14].

### Inclusion and exclusion criteria

The review included quantitative studies as well as qualitative and mixed-methods studies, also sourced from grey literature such as dissertations. Publications considering people with DM and diabetes-related information-seeking behaviour were included that used the following terms in different combinations and their synonyms, e.g. information-seeking behaviour and/or information seeking, information search and/or seek for information.

Studies published in languages other than English or German were excluded, as well as letters, short reports, editorials, comments and discussion papers. However, they were used to find further studies.

There were no exclusion criteria concerning the type of diabetes or the assessment tools used to collect data about information-seeking behaviour. None of the studies were excluded because of their low quality.

### Study selection process

A pre-test for the title and abstract screening was performed, which included 100 articles selected by three reviewers. Potentially eligible publications were selected by their title and abstract and categorised into ‘included’, ‘unclear’ and ‘excluded’. Literature identified by title and abstract and labelled as ‘included’ or ‘unclear’ was screened as full texts and analysed for final inclusion. Two raters reviewed independently each step and a third reviewer resolved unclear coding.

### Data extraction and synthesis

#### Data extraction

Data extraction was performed according primarily to Wilson’s ‘model of information behaviour’ as described above (Table 1).

**Table 1:** Main categories of information-seeking behaviour according to Wilson’s model

Deductive categories of information-seeking behaviour (Wilson’s model)		
Main categories	Subcategories	Definition [8]
Types of information-seeking behaviour	Passive attention	‘Such as listening to the radio or watching television programmes, where information acquisition may take place without intentional seeking’
	Passive searching	‘Signifies those occasions when one type of search (or other behaviour) results in the acquisition of information that happens to be relevant to the individual’
	Active searching	Active searching is ‘where an individual actively seeks out information’
	Ongoing searching	‘Where active searching has already established the basic framework of knowledge, ideas, beliefs or values, but where occasional continuing search is carried out to update or expand one’s framework’
Intervening variables	Psychological	Psychological intervening variables include, e.g. cognitive dissonance, cognitive and emotional characteristics
	Demographic	Demographic intervening variables cover, e.g. age and sex
	Role-related or interpersonal	Role-related or interpersonal intervening variables cover, e.g. social systems, requirements and level of responsibility
	Environmental	Environmental intervening variables cover, e.g. time, geography and national cultures
	Source characteristics	Source characteristics cover, e.g. access, credibility and the channel of communication

However, two adjustments were made: (1) since information sources are implicitly described in the definitions of information-seeking behaviour [7], it was defined as an additional main category (instead of an associated variable). The preferred sources for gaining information, such as the Internet, television and health professionals, were subdivided. (2) A further main category was also introduced, namely content of information, since it is one of the main questions of the review.

#### Data synthesis

Data synthesis (narrative synthesis) was performed in accordance with the Cochrane methods for data analysis and syntheses [15], which is in line with the “Best-fit” framework synthesis as described by Carroll et al. [16], except the adjustment of the model, which was not the aim of our review.

Qualitative and quantitative data, including data from mixed-methods studies, were described, analysed separately and grouped. The characteristics of the studies and important differences between them were described systematically. The results were then tabulated. Relevant topics were highlighted and data transformed into a descriptive format. The results from the content analysis were described by summarising similar findings regarding information-seeking behaviour and associated variables from qualitative, quantitative and mixed-methods studies. Deductive and inductive content analyses were performed [17, 18]. Deductive categories were derived from Wilson’s model (Table 1), including the adjustments, and inductive categories or subcategories from the included publications. Several steps were performed for the inductive approach [17, 18]. The first step was to identify relevant data segments from the included studies. The second step was to develop data matrices including raw data, first codes and memos. The third step was to arrange similar memos together and to find categories into which the corresponding segments fitted. This step was repeated and re-evaluated several times. A combined coding protocol and data extraction sheet was developed for the deductive and inductive categories.

#### Critical appraisal and risk of bias

Qualitative and quantitative studies were analysed using the quality criteria of the National Institute for Health and Care Excellence (NICE) [19], using a coherent set of critical appraisals. These NICE appraisal checklists were developed to assess the risk of bias in diverse types of studies [19]. The degree of bias risk can be determined at the end of the appraisal process.

Quality criteria include factors such as whether the study population is well described and whether it is representative, how explanatory variables were selected, how the confounding factors were identified and controlled, whether the outcome measures were reliable or complete, whether the power was calculated and multiple explanatory variables were considered, whether the precision of the association was provided, and whether the studies were internally or externally valid for quantitative studies [19]. For qualitative studies, quality criteria regarding the methods were, for example, whether the qualitative approach was appropriate, the study aim was clear, the research design and methodology were defensible, how well data collection was performed, whether the researchers’ role and the context were clearly described, whether the methods were reliable, and whether the data analysis was sufficiently rigorous as well as reliable. Additional criteria for apprising the results and conclusions were, for example, whether the data was rich, relevant, the findings convincing and the conclusions adequate [19]. According to NICE grading, the study’s quality was described as follows: ‘(++) all or most of the checklist criteria have been fulfilled, where they have not been fulfilled the conclusions are very unlikely to alter; (+) some of the checklist criteria have been fulfilled, where they have not been fulfilled, or not adequately described, the conclusions are unlikely to alter; (-) few or no checklist criteria have been fulfilled and the conclusions are likely or very likely to alter’ [19].

Criteria from the “Mixed Methods Appraisal Tool (MMAT)” for mixed-methods studies include whether the mixed-methods design was appropriate, the integration was relevant to address the research question (objective) and whether the consideration given to the limitations associated with this integration, e.g. the divergence of qualitative and quantitative data (or results) in a triangulation design, was appropriate [20, 21]. The study quality was described by fulfilled or not fulfilled criteria [20, 21]. Two raters performed independently the critical appraisal and then resolved differences, if necessary.

## Results

**Fig. 1:**
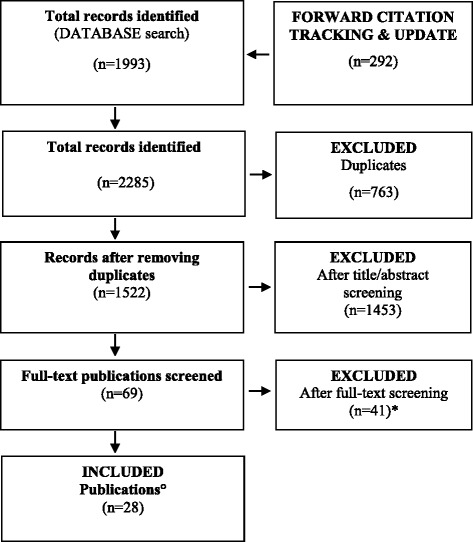
PRISMA flow diagram

A total of 2285 hits were identified. Finally, 28 publications (covering 26 studies) were included, of which 17 studies (covering 18 publications) were found by searching for studies published up to June 2015 [5, 9, 10, 12, 13, 22–34] and two publications [35, 36] by forward citation tracking of which one study was new [36] and one was already identified in the first search process. Additionally, eight further studies (covering eight publications) were identified by updating the search in July 2017 [37–44] (see Fig. 1). The 26 selected studies comprise the following: one by forward citation tracking, 10 applied qualitative methods [9, 27–32, 37, 39, 40], 12 applied quantitative methods [5, 10, 22–26, 36, 38, 41, 42, 44] and four mixed-methods [12, 33, 34, 43].

The studies investigated information-seeking behaviour, applied for example semi-standardised questionnaires [32, 34, 37, 43], interviews with open-ended questions [12, 28, 30, 31, 33, 39, 43] and focus groups [9, 27, 29, 31, 40].

The sample sizes of the participants with DM of the studies ranged from *n* = 12 [40] to *n* = 3652 [41]. Most of the studies (*n* = 25) provided information about gender, age and the region participants were recruited from. Three studies [34, 39, 42] included only female participants because they focussed on DM and pregnancy. Most of the studies included adults, and only one focused on younger people with DM [23]. Participants were mostly recruited from Europe (*n* = 10) [5, 22–24, 29, 31, 34, 37, 38, 43] or North America (*n* = 6) [9, 12, 25, 27, 30, 33], while five studies took place in Asia [26, 28, 36, 40, 44], three in Australia [39, 41, 42] and one in Canada [10]. The region of one study is unknown [32]. An overview of the included studies is presented in Table 2.

**Table 2:** Overview of the identified studies

Author/year	Design/method	Recruitment setting	Sample size	Population				Study focus	Findings	Critical appraisal	Number of criteria*
				Age	Sex	Type of DM** (and duration)	Region				
Quantitative studies											
Enwald et al. 2012 [22]	Cross-sectional study (questionnaire within an experimental study)	Register of the University of Oulu, medical records of health centres	*n* = 72	Mainly > 60	f, m	Risk of T2D (defined as pre-diabetes)	Finland	Relation between physiological measurements (BMI, fitness level) and information needs and information behaviour	BMI and fitness level of pre-diabetic patients are associated with information-seeking behaviour	+	2pp, 810p, 0m, 0, NR, 9NA
Giménez-Pérez et al. 2015 [38]	Cross-sectional study (questionnaire)	Endocrinology unit of a university hospital	*n* = 289	Average 43	f, m	T1D for at least 1 year	Spain	Health-related use of Internet technologies	Use of new Internet technologies among patients with T1D is low, e-mail preferred channel of communication with HCP	+	2pp, 8p, 0m, 0NR, 9NR
Hyman et al. 2012 [10]	Cross-sectional study (questionnaire)	Poster, community health centre, DM education centre, specialised clinic, Canadian Diabetes Association	*n* = 184	Average: immigrants 51.2, Canadian-born 52.3	f, m	Self-reported T2D	Toronto, Canada (Toronto)	Self-management, health service use and information-seeking behaviour of recent immigrants and Canadian-born	Differences in performing self-management (regular blood glucose and foot checks) and perception of health service between immigrants and Canadian-born	+	3pp, 68p, 0m, 1NR, 9NA
Jamal et al. 2015 [36]	Cross-sectional study (questionnaire)	Unknown	*n* = 344	Adults (> 16 years old)	f, m	T2D	Riyadh, Saudi Arabia (Riyadh)	Online health information-seeking behaviour of people with T2D	Physicians and television preferred sources	+	4pp, 53p, 04m, 21NR, 9NA
Kalantzi et al. 2015 [5]	Cross-sectional study (questionnaire)	Outpatient clinic	*n* = 203	Adults (> 18 years old)	f, m	T1D, T2D	Athens, Greece (Athens)	Information-seeking behaviour of people with DM, information needs, Internet use, obstacles to information seeking	Diet and complication most important needs, physician preferred source, Internet not that important, most frequently barriers mentioned are costs and lack of time	+	2pp, 78p, 1m, 0NR 9NA
Lui et al. 2014 [41]	Baseline phase of a longitudinal study (questionnaire)	Australian government initiative	*n* = 3652	56–70	f, m	T2D	Queensland, Australia (Queensland)	Correlation between health and social characteristics and Internet use	Internet use associated with age, socioeconomic characteristics, duration, poor metabolic control and comorbidities	+	4pp, 6p, 0m, 0NR, 9NA
Nordfeldt et al. 2005 [23]	Cross-sectional study (questionnaire)	Paediatric clinics	*n* = 90	5–20	f, m	T1D for at least 1.5 years	Sweden	Internet health information-seeking behaviour of children and adolescents with T1D, motivation, satisfaction	Many use Internet for health information seeking and share it with others. ‘Searchers’ with shorter duration. Need for more and better Internet information	+	4pp, 64p, 0m, 2NR, 9NA
Robertson et al. 2005 [24]	Cross-sectional study (questionnaire)	Diabetes centres	*n* = 70	16–79	f, m	T1D, T2D	UK (Glasgow)	Information source of people with DM, satisfaction	Verbal information from healthcare professional is preferred, Internet use connected with age and educational level	+/-	0pp, 6p, 5p, 2p/m, 2m, 13NR, 9NA
Sayakhot and Carolan-Olah 2016 [42]	Cross-sectional study (questionnaire)	Diabetes clinic	*n* = 116	18–43	f	GDM	Victoria, Australia (Victoria)	Information sources and satisfaction of women with GDM	HCP, diabetes groups and Internet preferred sources. Correlation between age and place of birth and Internet use. Mostly satisfied with process of diagnosis	+	2pp, 7p, 0m, 0NR, 9NA
Shaw and Johnson 2011 [25]	Cross-sectional study (questionnaire)	Flyers in primary care clinics and libraries	*n* = 57	Adults (> 21 years)	f, m	T2D	USA (Sub-urban, rural south-eastern)	Online health information-seeking behaviour of people with DM	Majority use Internet for health information seeking, many use social networks like Facebook or MySpace and discuss in chats	-	0pp, 65p, 46m, 0NR 9NA,
Yamamoto et al. 2011 [26]	Cross-sectional study (questionnaire)	Diabetes clinics	*n* = 137	20–75	NR	T1D for at least 6 months	Japan	Information about islet transplantation of people with T1D, associated factors, sources	Main sources are magazines and broadcast media, physician is preferred source, but mostly not able to give information about islet transplantation	+	4pp, 57p, 1m, 0NR, 9NA
Zare-Farashbandi et al. 2016 [44]	Cross-sectional study (questionnaire)	10 health centres under the supervision of the Deputy of Health of Isfahan Province	*n* = 362	20–82	f, m	Risk of T2D (defined as pre-diabetes), GDM, T2DM	Iran (Isfahan)	Effect of contextual factors on the health information-seeking behaviour of people with diabetes	An association between the time passed since diagnosis and information-seeking behaviour.	+	0pp, 7p, 0m, 2NR, 9NA
Qualitative studies											
Connolly and Crosby 2014 [27]	Focus group	Qualified health centre	*n* = 25	Average 54	f, m	Not defined	Hawaii	e-health literacy of individuals from a medically underserved area in Hawaii	Low e-health literacy level, often access to Internet without use for health information seeking, often ability to handle when information missing	+	8/14
Fergie et al. 2015 [37]	Interview	Online, organisations for young adults, other participants	*n* = 20 T2DM *n* = 40 people with common mental health disorders	18–30	f, m	Not defined	UK (Glasgow), UK	Online information-seeking behaviour of young people with DM or common mental health disorders	Internet preferred source of information for many participants, different between professionally produced and social media sites	+	12/14
Kilgour et al. 2015 [39]	Interview	Tertiary referral hospital	*n* = 13	29–41	f	GDM	Australia (Queensland), Australia	Postnatal follow-up and communication experiences of women with GDM	Need for accurate information and possibility to discuss information with HCP	++	13/14
Longo et al. 2010 [9]	Focus group (5–8 participants each session)	Clinic	*n* = 46	48–77	f, m	T1D, T2D	American Midwestern city	Health information-seeking and use, information source, active seeking and passive seeking	Passive attainment of information important, Internet for active seeking, relationships a and healthcare professionals help to understand information	+	12/14
Low et al. 2016 [40]	Interview, focus group	Public and private primary care clinics	*n* = 12 *n* = 9 family member *n* = 5 Health care professionals	50–62	f, m	T2D	Malaysia	Influence of social networks on help-seeking behaviour of people with T2D	Important influences from family, friends, HCP	++	13/14
Meyfroidt et al. 2013 [29]	Focus group (6 groups)	Community health centre, solo and group practices	*n* = 21	41–85	f, m	T2D	Belgium (Brussels)-Capital region of Belgium	Seeking and use of information sources of people with DM, active and passive seeking over time	General practitioner most important source, healthcare professionals most reliable	++	14/14
Milewski and Chen 2010 [30]	Interview	Outpatient clinic, flyers	*n* = 19	NR	f, m	T2D	USA (Southern California)	Information-seeking behaviour of people with DM, barriers of information use	5 barriers identified: ‘Motivation fade over time’, ‘Passively Seeking Information’, ‘Inconsistency of Information’, ‘Generality of Information’, ‘Loss of Information’	+	11/14
Moonaghi et al. 2014 [28]	Interview	NR	*n* = 15	Average 51	f, m	T2D for at least a year	Tabriz, Iran (Tabriz)	Health information-seeking behaviour of Iranian DM patients	Social context important for decision-making and information seeking behaviour	++	13/14
Newton et al. 2012 [31]	Interview (*N* = 25), focus group (*N* = 12), questionnaire (*N* = 6)	DM support group	*n* = 37	Mainly > 60	f, m	T2D	England/UK (Inner London district)	Information seeking and use of mainly older people with DM from a structurally lacking area, motivation, sources	Seeking and use influenced by social resources and context, which are important for effective and high quality care. Second most important factor is duration of disease	+	9/14
Wilson 2013 [32]	Survey (questionnaire)	Email of insulin pump therapy group	*n* = 30	22–64	f, m	T1D, T2D	UK (Glasgow)	Internet health information seeking of people with long-term DM	Internet used for general questions, healthcare professionals for more specific needs	-	5/14
Mixed-methods studies											
Morgan and Trauth 2013 [33]	Interviews	Database of Pennsylvania State University Institute for Diabetes and Obesity, investigator contacts	*n* = 30	Adults (> 18 years)	f, m	T1D, T2D for at least a year	USA (Central Pennsylvania and Southern Maryland)	Online health-information seeking and the demographic influence using a theoretical model	Seeking behaviour influenced by different factors such as access to healthcare providers, seeking success or the social network	+	9/21 (8NA, 1NR)
Sparud-Lundin et al. 2011 [34]	Survey (questionnaire)	Antenatal clinics	*n* = 105	30–36	f	T1D	Sweden	Online health information-seeking behaviour, use and information needs of childbearing women, expectations for future online possibilities	Many women with T1D seek health information online, particularly during pregnancy, precise expectations of web-based support	+	8/21 (8NA)
St Jean 2012 [12]	Questionnaire, interviews, card-sorting techniques	University websites, flyers at clinics and support group meetings	*n* = 34	32–81	f, m	T2D	USA, (Michigan)	Information behaviour of people with DM, associated factors, that facilitate or hinder their diabetes-related information seeking and use	Participants often did not know their information needs until they found information about it. Some mentioned avoidance in the beginning. Different factors, time included, influencing information seeking behaviour	++	11/21 (8NA)
St Jean 2014 [13]	Questionnaire, interviews, card-sorting techniques	University websites, flyers at clinics and support group meetings	*n* = 34	32–81	f, m	T2D	USA, (Michigan)	Information behaviour of people with DM, associated factors, that facilitate or hinder their diabetes-related information seeking and use	The new type of card-sorting technique was well accepted by the study participants. The combination of the card-sorting technique and think aloud protocol within this technique generated contextually rich data about people’s diabetes course.	+	7/21 (13NA)
St Jean 2016 [35]	Questionnaire, interviews, card-sorting techniques	University websites, flyers at clinics and support group meetings	*n* = 34	32–81	f, m	T2D	USA, (Michigan)	Information behaviour of people with DM, associated factors, that facilitate or hinder their diabetes-related information seeking and use	This study showed several types of factors (physical, social, affective, and cognitive) that may facilitate, hinder, or impede the health-related information seeking.	+	6/21 (12NA)
Weymann et al. 2016 [43]	Semi-structured interviews, questionnaire	University hospital, self-help groups, self-help associations	*n* = 10 (interviews) *n* = 178 (questionnaire)	36–86	f, m	T2D	Germany	Internet use, knowledge and information and support needs of people with T2D	Majority uses Internet, no correlation between age and Internet use, diabetes knowledge low, desire for shared decision-making	+	6/21 (8NA, 3NR)

Quantitative or qualitative studies and mixed-methods studies (following NICE grading):

‘(++) — all or most of the checklist criteria have been fulfilled, where they have not been fulfilled the conclusions are very unlikely to alter; (+) — some of the checklist criteria have been fulfilled, where they have not been fulfilled, or not adequately described, the conclusions are unlikely to alter; (-) — few or no checklist criteria have been fulfilled and the conclusions are likely or very likely to alter.’ (National Institute for Health and Care Excellence 2012)

pp: ‘Indicates that for that particular aspect of study design, the study has been designed or conducted in such a way as to minimise the risk of bias.’

p: ‘Indicates that either the answer to the checklist question is not clear from the way the study is reported, or that the study may not have addressed all potential sources of bias for that particular aspect of study design.’

m: ‘Should be reserved for those aspects of the study design in which significant sources of bias may persist.’

NR (not reported): ‘Should be reserved for those aspects in which the study under review fails to report how they have (or might have) been considered.’

NA (not applicable): ‘Should be reserved for those study design aspects that are not applicable given the study design under review (for example, allocation concealment would not be applicable for case–control studies).’Should be reserved for those aspects in which the study under review fails to report how they have (or might have) been considered.’

(National Institute for Health and Care Excellence 2012)

*T1D* type 1 diabetes, *T2D* type 2 diabetes, *GDM* gestational diabetes

Types of DM were specified in 23 studies: 11 studies included participants with type 2 diabetes mellitus (T2DM) [10, 12, 25, 28–31, 36, 40, 41, 43], four included participants with type 1 diabetes mellitus (T1DM) [23, 26, 34, 38], five included participants with T1DM and T2DM [5, 9, 24, 32, 33], two with gestational diabetes (GDM) [39, 42] and one study included participants with prediabetes [22]. One study included people with T2DM as well as people with prediabetes and GDM [44].

Five studies provided specifications about the minimum duration of the diagnosis of DM [23, 26, 28, 33, 38], and six studies provided information on HbA_1c_% [5, 12, 26, 38, 41, 43] and five on body mass index (BMI) [22, 26, 27, 39, 41].

Only four studies provided a clear definition of ‘information-seeking behaviour’. It is seen in the context of health information behaviour, consisting of ‘health-related information needs, information seeking, and information use’ [12]. Longo et al. describes it as consisting of ‘active information seeking’, with the aim of acquiring specific information for an intended purpose with a ‘passive receipt of information’ where information is gained ‘unintentionally’ [9]. Moonaghi et al. provides an extended explanation by describing it as being ‘influenced by peoples’ perceptions about a disease within the context of traditional and cultural beliefs and attitudes’. Furthermore, information seeking is outlined ‘as a key coping strategy in health-promotion activities and in the psychosocial adjustment to illness’ [28]. Zare-Farashbandi et al. defined that ‘Health information-seeking behaviours of a person include search, discovery, and use of information related to diseases, health-threatening factors, and health care’ [44].

The critical appraisal performed showed that only three of the 28 publications (covering 26 studies) identified fulfilled all or most of the checklist criteria of NICE or MMAT. In accordance with the NICE grading system, the other publications fulfilled some (*n* = 14) or a few (*n* = 5) of the quality criteria and displayed a higher level of bias [19] (Table 2).

### Information-seeking behaviour: type, sources and content

In total, five types of information-seeking behaviours were identified (Table 3), namely ‘passive attention’ (*n* = 4) [9, 28, 30, 44], ‘passive search’ (*n* = 2) [29, 30], ‘active search’ (*n* = 7) [9, 12, 28–31, 35, 44] and ‘ongoing search’ (*n* = 4) [9, 12, 28, 29] as defined in Wilson’s model and an additional ‘combined search types’ (*n* = 6) category [9, 12, 28, 29, 31, 32]. For example, one study reported that the information-seeking process began with a more general approach and became more specific [12].

**Table 3:** Information-seeking behaviour

Main category	Subcategory	Examples	Study designs		
			Qualitative studies	Quantitative studies	Mixed-methods studies
Types of information-seeking behaviour [9, 12, 13, 28–32, 35, 44]	Passive attention	Picking up information in the newspaper or TV shows [9]	[9, 28, 30]	[44]	–
	Passive search	Searching without a specific purpose; passively received information [29]	[29, 30]	–	–
	Active search	Active seeking in the beginning [29]	[28–31]	[9, 44]	[12, 35]
	Ongoing search	Looking for credible sources to ‘weave their ongoing web of information’ [9]	[9, 28, 29]	–	[12, 13]
	Combined types	Start with general information and continue with more specific ones [12]	[9, 28, 29, 31, 32]	–	[12, 13]
Information sources [5, 9, 10, 12, 13, 23–34, 36–43]	Healthcare professional	Physicians, nurses, dietitians, diabetes educator, pharmacists [12]	[9, 27, 29–32, 37, 39, 40]	[5, 10, 24, 26, 36, 42]	[12, 13, 33, 35, 43]
	Diabetes groups	Groups as one of the major information sources [31]	[31]	[42]	[12, 13]
	Relatives/friends	Family with DM history as an information source [29]	[9, 29, 31, 40]	[10, 26, 36]	[12, 13, 33, 43]
	Other patients	Other T1DM patients or patient society [26]	–	[26]	[12, 13]
	Internet	Superficial and particular websites [33]	[9, 27, 29, 31, 32, 37, 39, 40]	[5, 10, 23–26, 36, 38, 41, 42]	[12, 13, 33, 34, 43]
	Brochures/magazines	Printed media is still a mentioned source [9]	[9, 29, 31, 40]	[26, 36]	[12, 13, 35]
	Books	Books as a helpful source, especially obtain information after diagnosis [33]	[9, 29, 31, 40]	–	[12, 13, 33]
	Broadcast media	Radio and television [5]	[29]	[5, 26, 36]	[12, 13, 43]
	Social networks	Facebook or twitter [9]	[30, 37, 40]	[25, 36, 38]	–
Content of information [5, 12, 13, 22–24, 32, 34, 36, 37, 39, 43]	Diet	Information need of pre-diabetic patients about nutrition [22]; alcohol and DM [32]	[32]	[5, 22, 23, 36]	[12, 13, 34, 43]
	Complications	Symptoms of DM kidney failure [32]	[32]	[5, 23, 24, 36]	[12, 13, 34, 43]
	Exercise	Effect of exercise on blood sugar level [12]	[32, 37]	[5, 22, 23]	[12, 13, 34, 43]
	Medication and pharmacological interactions	Insulin treatment [34]	[32]	[23, 36]	[12, 13, 34, 43]
	Pregnancy	Breastfeeding and DM [34]	–	[23, 39]	[34]

Nine inductively developed subcategories of information sources were addressed in the studies. The most frequently reported sources of information were the Internet (*n* = 22) [5, 9, 10, 12, 23–27, 29, 31–34, 36–43] and healthcare professionals (*n* = 17) [5, 9, 10, 12, 24, 26, 27, 29–33, 36, 37, 39, 40, 42, 43]. Slightly fewer studies mentioned ‘relatives and friends’ (*n* = 10) [9, 10, 12, 26, 29, 31, 33, 36, 40, 43], ‘brochures and magazines’ (*n* = 7) [9, 12, 26, 29, 31, 36, 40], ‘books’ (*n* = 6) [9, 12, 29, 31, 33, 40] and ‘broadcast media’ (*n* = 6) [5, 12, 26, 29, 36, 43] as a source of information. Social networks were identified as information sources in six studies [25, 30, 36–38, 40], diabetes groups in three [12, 31, 42] and other patients [12, 26] in two studies. One study addressed several information sources [44]. However, these were described as “traditional” or “novel” sources, for example, without further specification of the terms [44].

Regarding the content of information, people with DM searched for ‘diet’ (*n* = 8) [5, 12, 22, 23, 32, 34, 36, 43], ‘complications’ (*n* = 8) [5, 12, 23, 24, 32, 34, 36, 43], ‘exercise’ (*n* = 8) [5, 12, 22, 23, 32, 34, 37, 43] and ‘medication and pharmacological interactions’ (*n* = 6) [12, 23, 32, 34, 36, 43]. Fewer studies investigated the information needs of pregnant women with DM (*n* = 3) [23, 34, 39].

### Associated variables

Associated variables regarding the information-seeking behaviour of people with DM are presented in Table 4 in their respective categories.

**Table 4:** Associated variables of information-seeking behaviour

Category of associated variable	Category of information-seeking behaviour	Specific associated variables	Study
Quantitative studies			
Demographic	Types of information-seeking behaviour	Older and female participants show reduced information seeking	[5]
	Information sources	Younger participants often use Internet to find health information	[5, 24, 36, 38, 41]
		Male participants prefer ophthalmologists	[5]
		Male and younger participants prefer broadcast media as a source	[5]
		Female uses Internet more often for health information	[38]
		Older participants prefer Internet as a source	[42]
		Participants born outside Australia prefer Internet as a source	[42]
		Immigrants (Canadian) prefer family and friends as a source of information	[10]
		Immigrants (Canadian) use physicians less than Canadian-born	[10]
	Content of information	A younger age is related to searching for information about exercise	[5]
		A younger age is related to searching for complications	[5]
		A younger age is related to searching for information about hypoglycaemia	[5]
		A younger age is related to searching for dietary issues	[5]
Role-related/interpersonal	Types of information-seeking behaviour	People with DM with a family history of DM have significantly higher average information-seeking behaviour scores of active information receipt and interpersonal relationships	[44]
	Information sources	Receiving information from people and from novel media, and the effect of information according to the patient, was significantly higher for people with diabetes during pregnancy compared with prediabetes and diabetes.	[44]
Source characteristics	Information sources	Unorganised information appears to act as a barrier for younger people with diabetes	[5]
Socioeconomic	Types of information-seeking behaviour	Lower education level is related to reduced seeking behaviour	[5]
		Participants with a lower income show reduced information seeking	[5]
	Information sources	Higher education level is related to a preference for a combination of verbal and written information	[24]
		Higher education level is related to Internet use	[24, 36, 41]
		Particularly, patients with lower education level prefer physicians as their main source of information	[5]
		Lower education level is related to a preference for verbal communication	[24]
		A higher income is related to Internet use	[5, 36, 41]
		A lower income is related to a preference for physicians as the main source of information	[5]
	Content of information	Higher education level is related to information needs about complications	[5]
		Higher education level is related to information needs about hypoglycaemia	[5]
		Higher education level is related to information needs about exercise	[5]
		A higher income is related to information needs about complications	[5]
		A higher income is related to information needs about exercise	[5]
Duration of DM	Types of information-seeking behaviour	Longer duration of DM is related to reduced seeking	[5, 44]
	Information sources	Longer duration of DM is related to preference for ophthalmologists	[5]
		Shorter duration of DM is related to Internet use	[5, 36, 41]
	Content of information	Shorter duration of DM is related to information needs about exercise	[5]
		Shorter duration of DM is related to information needs about hypoglycaemia	[5]
		Longer duration of DM is related to information needs about foot complications	[5]
Lifestyle	Information sources	Human information behaviour is related to BMI and fitness level: lower self-reported fitness level and higher BMI are related to higher desire for tailored information	[22]
		Higher BMI is related to Internet use	[41]
	Content of information	Higher BMI is related to information seeking about nutrition	[22]
Qualitative studies			
Demographic	Information sources	Different demographic factors, e.g. gender	[28]
		In an older population, the minority use the Internet for health information seeking	[27]
Role-related/interpersonal	Information sources	Family as a motivator for using the Internet for health information seeking	[27]
Environmental	Information sources	Cultural aspects like a preference for herbal medicine or religious and spiritual beliefs could lead to an avoidance of healthcare professionals	[28]
Source characteristics	Types of information-seeking behaviour	Lower quality could maybe lead to less active seeking	[30]
Socioeconomic	Types of information-seeking behaviour	Higher education level is related to active seeking and a complex style of information-seeking behaviour	[28, 31]
		A higher income is related to a use of a more complex style of information-seeking behaviour	[31]
		A lower income is related to a use of simple styles of information-seeking behaviour	[31]
	Information sources	Higher education level is related to Internet use	[29]
Duration of DM	Types of information-seeking behaviour	Shorter duration of DM is related to passive seeking	[29]
		Short duration of DM is related to active seeking; longer duration leads to less active seeking	[30]
		Short duration of DM shows a search for baseline information and after a longer duration of DM more complex information seeking starts	[31]
	Information sources	Health professionals consulted in all phases of disease	[9, 29]
Mixed-methods studies			
Demographic	Information sources	Black participants mainly obtain baseline information from a physician	[33]
Role-related/interpersonal	Content of information	For childbearing women, information about pregnancy and DM are most important	[34]
Source characteristics	Types of information seeking	Doctor motivates active seeking of information	[35]
		Trust as a factor influencing the use of Internet as a source	[33]
Socioeconomic	Types of information-seeking behaviour	Lower education level is related to reduced seeking behaviour and the perception of importance to learn more about DM	[12]
	Information sources	Higher education level is related to Internet use	[34]
		Lower education level is related to the use of special websites	[33]
Duration of DM	Types of information-seeking behaviour	Short duration of DM is related to active seeking; longer duration leads to less active seeking	[12]

Seven main categories associated with information-seeking behaviour were identified in the selected studies. Four categories were in line with Wilson’s model, namely ‘demographic’ (*n* = 10) [5, 10, 24, 27, 28, 33, 36, 38, 41, 42], ‘personal and role-related/interpersonal’ (*n* = 3) [27, 34, 44], ‘environmental’ (*n* = 1) [28], and ‘source characteristics’ (*n* = 4) [29, 30, 33, 35]. Variables of the category ‘psychological’ as defined in Wilson’s model were not addressed in the selected studies. Three further categories were ‘socioeconomic’ (*n* = 11) [5, 12, 24, 28, 29, 31–34, 36, 41], ‘duration of diabetes’ (*n* = 9) [5, 9, 12, 29–31, 36, 41, 44] and ‘lifestyle’ (*n* = 2) [22, 41].

Variables of the category ‘demographic’ associated with preferred information sources were identified in 10 studies [5, 10, 24, 27, 28, 33, 36, 38, 41, 42]. One study showed an association between female gender and older age, and reduced information-seeking behaviour [5]. Furthermore, studies showed that younger participants used the Internet more often to find health information [5, 24, 36, 38, 41] than the older population [27]. Only a few of the older participants used the Internet to seek health information [27]. Kalantzi et al. identified that younger participants preferred information about exercise, hypoglycaemia and dietary issues [5].

Variables of the category ‘personal, role-related and interpersonal’ were identified in three studies [27, 34, 44]. Childbearing women with T1DM preferred information about pregnancy and DM [34]. There appears to be a correlation between family and searching the Internet for health information [27]. Having a family history of DM is associated with a higher average of information-seeking behaviour scores, e.g. of interpersonal relationships [44].

Variables of the category ‘environmental’ were addressed in one study [28]. It was pointed out that cultural aspects such as a preference for herbal medicine or religious and spiritual beliefs could lead to an avoidance of healthcare professionals [28].

‘Source characteristics’ were also addressed [5, 29, 30, 33, 35]. The quality of information and its sources appears to be an associated variable. For example, Meyfroidt et al. point out that participants trusted information obtained from healthcare professionals most, due to their experience [29]. However, less trust is placed in advertisements, for example [29]. Milewski and Chen demonstrate that information that often addressed patients with recently diagnosed DM, especially in flyers and brochures, did not meet individual information needs [30]. Furthermore, this study showed that lower-quality information could lead to less-active seeking [30]. Another study showed that unorganised information appears to act as a barrier for younger people with diabetes [5].

Variables of the category ‘socioeconomic’ containing the variables education and income were identified in 11 studies [5, 12, 24, 28, 29, 31–34, 36, 41]. A higher level of education is associated with active information seeking and with more complex styles of information-seeking behaviour [28, 31]. Additionally, eight studies show that there is a correlation between educational background and preference for specific information sources [5, 24, 29, 33, 34, 36, 41]. A higher level of education seems to be associated with Internet use [24, 29, 34, 36, 41], while participants with a lower educational background appear to prefer verbal communication [24]. A higher income seems to be associated with the use of more complex styles of information-seeking behaviour, while participants with a lower income displayed more simple styles of information-seeking behaviour [31]. Similarly, two studies showed an association between socioeconomic variables and a preference for specific information sources [5, 41]. Kalantzi et al. show that a higher income is associated with a preference for the Internet as an information source, while a lower income is associated with a preference for personal contact (e.g. physicians) as the main source of information [5]. One study identified that a specific content of information is associated with a higher income and education level [5]. Participants with a higher level of education expressed greater interest in information about complications, hypoglycaemia and exercise. Similarly, participants with a higher income preferred information about complications and exercise [5].

Variables of the category ‘duration of DM’ were found in nine studies [5, 9, 12, 29–31, 36, 41, 44]. Five of them showed a correlation between the type of information-seeking behaviour and the duration of DM [5, 12, 29–31]. Two studies showed that participants start with active information seeking at the beginning of their disease, while a longer duration led to less-active seeking [5, 30]. Milewski and Chen assume that some participants seem to misjudge their disease as being stable and stop adapting once they have basic knowledge [30]. A correlation between duration of DM and preferred information sources was found in five studies [5, 9, 29, 36, 41], e.g. that the Internet was used as a fast alternative at an earlier stage, while health professionals were consulted in all phases of the disease [9, 29]. One study pointed out that participants in the first year of diagnosis preferred to search for baseline information, but after having DM for three or more years, they started to display more complex information-seeking behaviour [31].

Regarding the category ‘lifestyle’, Enwald et al. show that there is an association between the variables BMI and fitness classification, and the content of information [22]. Fitness classification was represented on a 7-point Likert scale with a range from ‘very poor’ to ‘excellent’ [45]. Participants with a lower fitness classification and a higher BMI showed a greater interest in seeking information about nutrition and exercise than those with better scores [22]. Lui et al. identified that participants with a higher BMI seem to search more often the Internet as a source of information [41].

## Discussion

To our knowledge, this is the first systematic review to analyse existing knowledge about information-seeking behaviour of people with DM. Wilson’s model proved to be suitable during the systematic review analysis. Overall, there were few studies with high heterogeneity regarding the research question, design, methods and participants.

### Types of information-seeking behaviour

Passive attention was described in patients with diabetes in some studies, in the sense that information was a by-product of everyday activities such as watching television or reading the newspaper [9]. The consequence of this passive role is that information does not always meet individual information needs [29–31]. Similar observations have not been made in people with cancer. However, a study with people with breast cancer indicated that healthcare professionals should pay attention to passive information seeking because the information obtained can influence healthcare decisions [46].

Some studies described more active information-seeking behaviour in which the Internet is used as an information source [9, 29]. Particularly, people with a higher level of education and higher income demonstrated a more active and complex type of information seeking [28, 31]. Moreover, a general shift from passive to more active seeking was recognised in one of our identified studies [5]. A similar observation was made in people with cancer [47]. It is therefore likely, then, that health professionals are dealing with more-informed patients [48, 49].

### Information sources

The Internet appears to be a preferred source of information, including special information [9, 23–25, 27, 29, 32, 37–43], especially for younger patients and those with a higher level of education [5, 24, 36, 38, 41]. Some participants even noted that they prefer more official websites, such as the guidelines of the UK National Institute for Health and Care Excellence, to their doctor’s opinion [37]. However, people with DM largely wish to have Internet information verified by their general practitioner or other healthcare professionals such as dieticians and medical specialists [9, 23, 29, 39], and they ranked professionals as an information source highly [5, 24, 36]. Furthermore, patients with other chronic diseases such as cancer ranked healthcare professionals as their preferred source of information, whereas the media, including the Internet, was ranked in the middle [46, 47, 49]. Healthcare professionals such as diabetes educators ought to support people with DM to identify qualitative information and help them to improve their health literacy skills according to their individual needs [50].

### Content of information

The studies showed that people with diabetes have a need to search for information about diabetes treatment [5, 12, 13, 22–24, 32, 34, 36, 37, 43]. However, it remains unclear, if information meets their information needs. It can be assumed that the information available does not meet individual information needs [29, 30] or that additional information is needed in where the disease has progressed [12, 28]. Also, a systematic review showed that people with cancer can have a deficit of information regarding ‘treatment options’ and ‘side effects of treatment’ [47].

### Associated variables

Individual characteristics (e.g. demographics, socioeconomic status) and the duration of the disease appear to be associated with information-seeking behaviour [5, 10, 12, 24, 27–29, 31, 38, 41, 42, 44]. Carlsson found similar results with people with cancer: there was a significant correlation between a higher level of education and an active type of seeking behaviour, and younger participants used the Internet to find health information more often than older participants [51]. Interestingly, patients who predominantly obtain information from the Internet tend to make more independent health decisions or determine whether they need professional support. The Internet is also used where people are dissatisfied with the information provided by health professionals [48]. The progression of the disease is related to seeking behaviour, e.g. a reason for active search [12, 36], and for contacting a doctor [28]. Other studies, for example those including people with end-of-life diabetes, found that there is a correlation between individual information needs, especially those of informal caregivers, and emotional aspects, with the trajectory of the disease [52].

### Implications

Our findings indicate that demographics and the socioeconomic status of people with DM are relevant in information management. It transpires that young people with DM require help from professionals in verifying Internet-based information, and perhaps additional information, while more detailed information ought to be provided to older patients and patients with a lower level of education, preferably verbally [24]. Furthermore, health professionals appear to be confronted with a shift from a more passive to a more active patient who is influenced by information from the Internet [9, 23, 25, 27, 29, 32, 48]. Perhaps, a more comprehensive way of exchanging information is needed to help patients to identify reliable information on the Internet by presenting suitable databases and preparing them to deal with several types of information.

Information provision could consider the stage of the disease [28, 36, 52], and informal caregivers could be involved where the disease is in a progressed stage. Research is needed to investigate individual strategies and needs in information-seeking behaviour over the course of the disease, giving consideration to demographics, socioeconomic status and the type of seeking. The heterogeneity of the studies’ populations indicates that further research is needed for each type of diabetes, also with regard to cultural aspects in information-seeking behaviour. The results of the critical appraisal also indicate that there is a need for well-designed studies with a higher level of internal and external validity.

### Limitations

The inclusion criteria were handled rigorously, resulting in the inclusion of a small number of studies. A selection bias can be assumed because of the language and database restrictions. Studies published up to June 2015 were searched. In September 2016 and in June 2017, a forward citation tracking was performed in Google Scholar to find current relevant studies by searching studies that cited already-identified core publications [14]. We also performed an update of two of our core databases (MEDLINE, CINAHL) and identified several new publications. However, it cannot be completely ruled out that some publications were missed after June 2015.

## Conclusion

There are a low number of studies analysing information-seeking behaviour, with a high heterogeneity regarding the research question, design, methods and participants. Both passive and active seeking seem to be performed by the patients; however, there may be a shift towards more active information-seeking behaviour. There is an association between information-seeking behaviour and demographics, socioeconomic and environmental aspects, source characteristics, and individual needs, including variations due to the progression of the disease. Younger people with higher levels of education and higher incomes especially prefer to search for information on the Internet, which is, however, not a substitute for information provided by healthcare professionals. More well-performed studies are needed to re-evaluate existing models of patient information-seeking behaviour.

## Additional files

Additional file 1: The PRISMA statement (2009). (DOC 84 kb)

Additional file 2: Update search strategy (MEDLINE, CINAHL) and search strategy (Ia and Ib). (DOC 343 kb)

## Abbreviations

BMI: Body mass index
CINAHL: Cumulative Index to Nursing and Allied Health Literature
DARE: Database of Abstract of Reviews of Effects
DDZ: German Diabetes Center
DM: Diabetes mellitus
DZD: German Center for Diabetes Research
EED: The NHS Economic Evaluation Database
EMBASE: Excerpta Medica Database
GDM: Gestational diabetes
HbA_1C_%: Percentage of glycated haemoglobin
Iddm: Insulin-dependent diabetes mellitus
Isb: Information-seeking behaviour
ISI: Institute of Science Index
MEDLINE: Medical Literature Analysis and Retrieval System Online
MMAT: Mixed Methods Appraisal Tool
NICE: National Institute for Health and Care Excellence
Niddm: Noninsulin dependent diabetes mellitus
PRISMA: Preferred Reporting Items for Systematic Reviews and Meta-Analyses
PsycINFO: Psychological Information Database
T1DM: Type 1 diabetes mellitus
T2DM: Type 2 diabetes mellitus
