# Correction to: Diabetes-related information-seeking behaviour: a systematic review

**DOI:** 10.1186/s13643-017-0646-9

**Published:** 2017-12-04

**Authors:** Silke Kuske, Tim Schiereck, Sandra Grobosch, Andrea Paduch, Sigrid Droste, Sarah Halbach, Andrea Icks

**Affiliations:** 1 Institute for Health Services Research and Health Economics, Centre for Health and Society, Faculty of Medicine, Heinrich Heine University Düsseldorf, Moorenstraße 5, 40225 Düsseldorf, Germany; 2 Department for Psychosomatic Medicine and Psychotherapy, Center for Health Communication and Health Services Research (CHSR), University Hospital Bonn, Sigmund-Freud-Str. 25, 53127 Bonn, Germany; 3 Institute for Health Services Research and Health Economics, German Diabetes Center, Düsseldorf, Germany; 4 German Center for Diabetes Research, Neuherberg, Germany

## Correction

During the production process for this article [1] some errors were introduced into Table 2. The correct version of Table 2 can be found below; the original article [1] has also been updated with the correct version of Table 2. BMC apologises to the authors and to readers for this error.

**Table 2:** Overview of the identified studies

Author/Year	Design/ Method	Recruitment setting	Sample size	Population				Study focus	Findings	Critical appraisal	Number of criteria*
				Age	Sex	Type of DM** (and duration)	Region				
Quantitative studies											
Enwald et al. 2012 [22]	Cross-sectional study (questionnaire within an experimental study)	Register of the University of Oulu, medical records of health centres	*n*=72	Mainly >60	f, m	Risk of T2D (defined as pre-diabetes)	Finland	Relation between physiological measurements (BMI, fitness level) and information needs and information behaviour	BMI and fitness level of pre-diabetic patients are associated with information-seeking behaviour	+	2pp, 8p, 0m, 0NR, 9NA
Giménez-Pérez et al. 2015 [38]	Cross-sectional study (questionnaire)	Endocrinology unit of a university hospital	*n*=289	Average 43	f, m	T1D for at least 1 year	Spain	Health-related use of Internet technologies	Use of new Internet technologies among patients with T1D is low, e-mail preferred channel of communication with HCP	+	2pp, 8p, 0m, 0NR, 9NA
Hyman et al. 2012 [10]	Cross-sectional study (questionnaire)	Poster, community health centre, DM education centre, specialized clinic, Canadian Diabetes Association	*n*=184	Average: Immigrants 51,2, Cana-dian-born 52,3	f, m	Self-reported T2D	Canada (Toronto)	Self-management, health service use and information-seeking behaviour of recent immigrants and Canadian-born	Differences in performing self-management (regular blood glucose and foot checks) and perception of health service between immigrants and Canadian-born	+	3pp, 6p, 0m, 1NR, 9NA
Jamal et al. 2015 [36]	Cross-sectional study (questionnaire)	University Medical City (teaching hospitals)	*n*=344	Adults (>16 years old)	f, m	T2D	Saudi Arabia (Riyadh)	Online health information-seeking behaviour of people with T2D	Physicians and television preferred sources	+	3pp, 5p, 0m, 2NR, 9NA
Kalantzi et al. 2015 [5]	Cross-sectional study (questionnaire)	Outpatient clinic	*n*=203	Adults (>18 years old)	f, m	T1D, T2D	Greece (Athens)	Information-seeking behaviour of people with DM, information needs, Internet use, obstacles to information seeking	Diet and complication are most important needs; the physician is a preferred source; Internet is not that important; most frequently barriers mentioned are costs and lack of time	+	2pp, 7p, 1m, 0NR 9NA,
Lui et al. 2014 [41]	Baseline phase of a longitudinal study (questionnaire)	Australian government initiative	*n*=3652	56-70	f, m	T2D	Australia (Queensland)	Correlation between health and social characteristics and Internet use	Internet use associated with age, socioeconomic characteristics, duration, poor metabolic control and comorbidities	+	4pp, 6p, 0m, 0NR, 9NA
Nordfeldt et al. 2005 [23]	Cross-sectional study (questionnaire)	Paediatric clinics	*n*=90	5-20	f, m	T1D for at least 1,5 years	Sweden	Internet health information seeking behaviour of children and adolescents with T1D, motivation, satisfaction	Many use internet for health information seeking and share it with others. ‘Searchers’ with shorter duration. Need for more and better Internet information	+	4pp, 4p, 0m, 2NR, 9NA
Robertson et al. 2005 [24]	Cross-sectional study (question-naire)	Diabetes centres	*n*=70	16-79	f, m	T1D, T2D	United Kingdom (Glasgow)	Information source of people with DM, satisfaction	Verbal information from healthcare professional is preferred, Internet use connected with age and educational level	+/-	0pp, 6p, 3m, 1NR, 9NA
Sayakhot and Carolan-Olah 2016 [42]	Cross-sectional study (questionnaire)	Diabetes clinic	*n*=116	18-43	f	GDM	Australia (Victoria)	Information sources and satisfaction of women with GDM	HCP, diabetes groups and Internet preferred sources; correlation between age and place of birth and Internet use; mostly satisfied with process of diagnosis	+	2pp, 8p, 0m, 0NR, 9NA
Shaw and Johnson 2011 [25]	Cross-sectional study (questionnaire)	Flyers in primary care clinics and libraries	*n*=57	Adults (>21 years)	f, m	T2D	USA (Sub-urban, rural south-eastern)	Online health information seeking behaviour of people with DM	Majority use Internet for health information seeking; many use social networks like Facebook or MySpace and discuss in chats	-	0pp, 6p, 4m, 0NR 9NA
Yamamoto et al. 2011 [26]	Cross-sectional study (questionnaire)	Diabetes clinics	*n*=137	20-75	NR	T1D for at least 6 months	Japan	Information about islet transplantation in people with T1D, associated factors, sources	Main sources are magazines and broadcast media; physicians are a preferred source of information, but mostly they do not have sufficient information about islet transplantation	+	4pp, 5p, 1m, 0NR, 9NA
Zare-Farashbandi et al 2016	Cross-sectional study (questionnaire)	Ten health centres under the super-vision of the Deputy of Health of Isfahan Province	*n*= 362	20-82	f,m	Risk of T2D (defined as pre-diabetes), GDM, T2DM	Iran (Isfahan)	Effect of contextual factors on the health information–seeking behaviour of people with diabetes	An association between the time passed since diagnosis and information-seeking behaviour.	+	0pp, 8p, 0m, 2NR, 9NA
Qualitative studies											
Connolly and Crosby 2014 [27]	Focus group	Qualified health centre	*n*=25	Average 54	f, m	Not defined	Hawaii	E-health literacy of individuals from a medically underserved area in Hawaii	Low e-health literacy level, often access to Internet without use for health information seeking, often ability to handle when information missing	+	8/14
Fergie et al. 2015 [37]	Interview	Online, organizations for young adults, other participants	*n*=20 T2DM n= 40 people with common mental health disorders	18-30	f, m	Not defined	United Kingdom (Glasgow)	Online information seeking behaviour of young people with DM or common mental health disorders	Internet preferred source of information for many participants; differences between professionally produced and social media sites	+	12/14
Kilgour et al. 2015 [39]	Interview	Tertiary referral hospital	*n*=13	29-41	f	GDM	Australia (Queensland)	Postnatal follow-up and communication experiences of women with GDM	Need for accurate information and possibility to discuss information with HCP	++	13/14
Longo et al. 2010 [9]	Focus group (5-8 parti-cipants each session)	Clinic	*n*=46	48-77	f, m	T1D, T2D	American midwestern city	Health information seeking and use, information source, active seeking and passive seeking	Passive attainment of information is important; Internet for active seeking, relationships and healthcare professionals help to understand information	+	12/14
Low et al. 2016 [40]	Interview, Focus group	Public and private primary care clinics	*n*=12 *n*=9 family member *n*=5 Health care professionals	50-62	f, m	T2D	Malaysia	Influence of social networks on help-seeking behaviour of people with T2D	Important influences from family, friends, HCP	++	13/14
Meyfroidt et al. 2013 [29]	Focus group (6 groups)	Community health centre, solo and group practices	*n*=21	41-85	f, m	T2D	Belgium (Brussels)	Seeking and use of information sources of people with DM, active and passive seeking over time	General practitioner is the most important source, healthcare professionals are most reliable	++	14/14
Milewski and Chen 2010 [30]	Interview	Outpatient clinic, flyers	*n*=19	NR	f, m	T2D	USA (Southern California)	Information seeking behaviour of people with DM, barriers of information use	5 barriers identified: ‘Motivation fade over time’, ‘Passively Seeking Information’, ‘Inconsistency of Information’, ‘Generality of Information’, ‘Loss of Information’	+	11/14
Moonaghi et al. 2014 [28]	Interview	NR	*n*=15	Average 51	f, m	T2D for at least a year	Iran (Tabriz)	Health information-seeking behaviour of Iranian DM patients	Social context important for decision making and information seeking behaviour	++	13/14
Newton et al. 2012 [31]	Interview (N=25), focus group (N=12), questionnaire (N=6)	DM support group	*n*=37	Mainly >60	f, m	T2D	England/UK (Inner London district)	Information seeking and use of mainly older people with DM from a structurally lacking area, motivation, sources	Seeking and use is influenced by social resources and context, which are important for effective and high quality care. Second most important factor is the duration of disease	+	9/14
Wilson 2013 [32]	Survey (questionnaire)	Email of insulin pump therapy group	*n*=30	22-64	f,m	T1D, T2D	United Kingdom (Glasgow)	Internet health information seeking of people with long-term DM	Internet used for general questions, healthcare professionals for more specific needs	-	5/14
Mixed-methods studies											
Morgan and Trauth 2013 [33]	Interviews	Database of Pennsylvania State University Institute for Diabetes and Obesity, investigator contacts	*n*=30	Adults (>18 years)	f, m	T1D, T2D for at least a year	USA (Central Pennsylvania and Southern Maryland)	Online health information seeking and the demographic influence using a theoretical model	Seeking behaviour is influenced by different factors such as access to healthcare providers, seeking success or the social network	+	9/21 (8NA, 1NR)
Sparud-Lundin et al. 2011 [34]	Survey (question-naire)	Antenatal clinics	*n*=105	30-36	f	T1D	Sweden	Online health information seeking behaviour, use and information needs of childbearing women, expectations for future online possibilities	Many women with T1D seek health information online, particularly during pregnancy, precise expectations of web-based support	+	8/21 (8NA)
St Jean 2012 [12]	Question-naire, interviews, card-sorting techniques	University websites, flyers at clinics and support group meetings	*n*=34	32-81	f, m	T2D	USA, (Michigan)	Information behaviour of people with DM, associated factors, that facilitate or hinder their diabetes-related information seeking and use	Participants often did not know their information needs until they found information about it. Some mentioned avoidance in the beginning. Different factors, time included, influencing information seeking behaviour	++	11/21 (8NA)
St Jean 2014 [13]	Question-naire, interviews, card-sorting techniques	University websites, flyers at clinics and support group meetings	*n*=34	32-81	f, m	T2D	USA, (Michigan)	Information behaviour of people with DM, associated factors, that facilitate or hinder their diabetes-related information seeking and use	The new type of card-sorting technique was well accepted by the study participants; the combination of the card-sorting technique and think aloud protocol within this technique generated contextually rich data about people's diabetes course	+	7/21 (13NA)
St Jean 2016 [35]	Question-naire, interviews, card-sorting techniques	University websites, flyers at clinics and support group meetings	*n*=34	32-81	f, m	T2D	USA, (Michigan)	Information behaviour of people with DM, associated factors, that facilitate or hinder their diabetes-related information seeking and use	This study showed several types of factors (physical, social, affective, and cognitive) that may facilitate, hinder, or impede the health-related information seeking	+	6/21 (12NA)
Weymann et al. 2016 [43]	Semi-structured interviews, questionnaire	University Hospital, self-help groups, self-help associations	*n*=10 (interviews) *n*=178 (questionnaire)	36-86	f, m	T2D	Germany	Internet use, knowledge and information and support needs of people with T2D	Majority uses internet, no correlation between age and internet use, diabetes knowledge low, desire for shared decision-making	+	6/21 (8NA, 3NR)

Quality rating (National Institute for Health and Care Excellence 2012):

‘(++) — all or most of the checklist criteria have been fulfilled, where they have not been fulfilled the conclusions are very unlikely to alter; (+) — some of the checklist criteria have been fulfilled, where they have not been fulfilled, or not adequately described, the conclusions are unlikely to alter; (-) — few or no checklist criteria have been fulfilled and the conclusions are likely or very likely to alter.’

Key criteria (National Institute for Health and Care Excellence 2012):

pp: ‘Indicates that for that particular aspect of study design, the study has been designed or conducted in such a way as to minimise the risk of bias.’

p: ‘Indicates that either the answer to the checklist question is not clear from the way the study is reported, or that the study may not have addressed all potential sources of bias for that particular aspect of study design.’

m: ‘Should be reserved for those aspects of the study design in which significant sources of bias may persist.’

NR (not reported): ‘Should be reserved for those aspects in which the study under review fails to report how they have (or might have) been considered.’

NA (not applicable): ‘Should be reserved for those study design aspects that are not applicable given the study design under review (for example, allocation concealment would not be applicable for case–control studies).’

T1D: Type 1 diabetes; T2D: Type 2 diabetes; GDM: Gestational diabetes
