# Supplementary material for: Diabetes-related information-seeking behaviour: a systematic review
Source: Syst Rev. 2017 Oct 24;6:212. doi: 10.1186/s13643-017-0602-8 (PMC5655894; doi:10.1186/s13643-017-0602-8)
Supplement: Supplementary file 1 — The PRISMA statement (2009). (DOC 84 kb) [file 13643_2017_602_MOESM1_ESM.doc]

**Appendix 1**

**PRISMA (Preferred Reporting Items for Systematic review and Meta-Analysis) 2009 checklist: recommended items to address in a systematic review protocol**

| Section and topic | Item No | Checklist item |
| --- | --- | --- |
| TITLE | | |
| Diabetes-Related Information-Seeking Behaviour: a systematic review | **1** | Identify the report as a systematic review, meta-analysis, or both. |
| ABSTRACT | | |
| **Background:** Information-seeking behaviour is necessary to improve knowledge on diabetes therapy and complications. Combined with other self-management skills and autonomous handling of the disease, it is essential for achieving treatment targets. However, a systematic review addressing this topic is lacking.  **Objective:** The aims of this systematic review were to identify and analyse existing knowledge of information-seeking behaviour: (1) types information-seeking behaviour, (2) information sources, (3) the content of searched information, and (4) associated variables that may affect information seeking behaviour.  **Method/Design:** The systematic review follows the PRISMA requirements. MEDLINE, CINAHL, EMBASE, ScienceDirect, PsycInfo, Cochrane Library, Web of Science, CCMed, ERIC, Journals@OVID, *Deutsches Ärzteblatt* and *Karlsruher virtueller Katalog* (KvK) databases were searched. Publications dealing with information-seeking behaviour of people with diabetes mellitus (DM) published up to June 2015 were included. A forward citation tracking was performed in September 2016 and June 2017. Additionally, an update of the two main databases (MEDLINE, CINAHL) was conducted, including studies published up to July 2017. Studies published in languages other than English or German were excluded, as well as letters, short reports, editorials, comments and discussion papers. A study selection and the critical appraisal of the selected studies were performed independently by two reviewers. A third reviewer was consulted if any disagreement was found. Data extraction and content analysis were performed using selected dimensions of Wilson’s ‘model of information behaviour’.  **Results:** Twenty-six studies were included. Five ‘types of information-seeking behaviour’ were identified, e.g. passive and active search. The ‘Internet’ and ‘healthcare professionals’ were the most frequently reported sources. ‘Diet’, ‘complications’, ‘exercise’ and ‘medications and pharmacological interactions’ were the most frequently identified content of information. Seven main associated variables were identified, including ‘socioeconomic’, ‘duration of DM’ and ‘lifestyle’.  **Discussion:** The systematic review provides a valuable overview of available knowledge on the information-seeking behaviour of people with DM, although there are only a few studies. There was a high heterogeneity regarding the research questions, designs, methods, and participants. Although the Internet is often used to seek information, health professionals still play an important role in supporting their patients’ information-seeking behaviour. Specific needs of people with diabetes must be taken into consideration. | **2** | Provide a structured summary including, as applicable: background; objectives;  data sources; study eligibility criteria,  participants, and interventions; study appraisal and synthesis methods; results; limitations; conclusions and  implications of key findings; systematic review registration number. |
| **INTRODUCTION** | | |
| **Rationale:**  Information-seeking behaviour is necessary to improve knowledge on diabetes therapy and complications. Combined with other self-management skills and autonomous handling of the disease, it is essential for achieving treatment targets. However, a systematic review addressing this topic is lacking. | **3** | Describe the rationale for the review in the context of what is already known |
| **Objectives**:  The aims of this systematic review were to identify and analyse existing knowledge of information-seeking behaviour: (1) types information-seeking behaviour, (2) information sources, (3) the content of searched information, and (4) associated variables that may affect information-seeking behaviour. | **4** | Provide an explicit statement of questions being addressed with reference to participants, interventions, comparisons, outcomes and study design (PICOS). |
| METHODS | | |
| **Protocol and Registration:**  In accordance with the guidelines, our systematic review protocol was registered with the International Prospective Register of Systematic Reviews (PROSPERO) on 4. April 2016 (CRD42016037312) | **5** | Indicate if a review protocol exists, if and where it can be accessed (e.g., Web address), and, if available, provide registration information including registration number. |
| **Eligibility criteria:**  The review included quantitative studies as well as qualitative and mixed-methods studies, also sourced from grey literature such as dissertations. Publications considering people with DM and diabetes-related information-seeking behaviour were included that used the following terms in different combinations and their synonyms, e.g. information-seeking behaviour and/or information seeking, information search, and/or seek for information.  Studies published in languages other than English or German were excluded, as well as letters, short reports, editorials, comments and discussion papers. However, they were used to find further studies.  There were no exclusion criteria concerning type of diabetes or the assessment tools used to collect data about information-seeking behaviour. None of the studies were excluded because of their low quality. | **6** | Specify study characteristics (e.g., PICOS, length of follow-up) and report characteristics (e.g., years considered, language, publication status) used as criteria for eligibility, giving rationale. |
| **Information sources:**  MEDLINE, EMBASE, CINAHL, ScienceDirect, The Cochrane Library, Web of Science, PsycINFO, CCMed, ERIC, and Journals@OVID. Additionally, national German sources were searched: *Deutsches Ärzteblatt* and *Karlsruher virtueller Katalog* (*KvK*). Studies published up to 01.07.2015 were considered. Additionally, an update of the two core databases (MEDLINE, CINAHL) was conducted, including studies published up to 26.07. 2017. | **7** | Describe all information sources (e.g., databases with dates of coverage, contact with study authors to identify additional studies) in the search and date last searched. |
| **Search:**  A full electronic search strategy is presented in the appendix. | **8** | Present full electronic search strategy for at least one database, including any limits used, such that it could be repeated. |
| **Study selection:**  A pre-test for the title and abstract screening was performed, which included 100 articles selected by three reviewers. Potentially eligible publications were selected by their title and abstract, and categorized into ‘included’, ‘unclear’ and ‘excluded’. Literature identified by title and abstract and labelled as ‘included’ or ‘unclear’ was screened as full texts and analysed for final inclusion. Two raters reviewed each step and a third reviewer resolved unclear coding. | **9** | State the process for selecting studies (i.e., screening, eligibility, included in systematic review, and, if applicable, included in the meta-analysis). |
| **Data collection process:**  Data extraction was performed according primarily to Wilson’s ‘model of information behaviour’ (Table 1). However, two adjustments were made: 1. since information sources are implicitly described in the definition of information-seeking behaviour, it was defined as an additional main category (instead of an associated variable). The preferred sources for gaining information, such as the Internet, television and health professionals, were subdivided. 2. A further main category was also introduced, namely content, since it is one of the main questions of the review. | **10** | Describe planned method of extracting data from reports (such as piloting forms, done independently, in duplicate), any processes for obtaining and confirming data from investigators. |
| **Data items:**  According to Wilson’s model, there are four different types of information-seeking behaviour: passive attention, passive or active searching, and ongoing search [1]. Passive attention is obtaining information without intending to look for it (e.g. watching television). Passive searching is finding relevant information while searching for other topics of information. This usually leads to active searching, ‘the principal mode’ in the process of information seeking, where ‘an individual actively seeks out information’ [1]. The last mode is ‘ongoing search’, which is performed during active search to update or to expand present information [1]. Besides types of information-seeking behaviour, Wilson’s model also defines information sources as associated variables and intervening variables, namely ‘psychological’, ‘demographic’, ‘role-related or interpersonal’, ‘environmental’ variables, and ‘source characteristics’ (e.g. currency, appropriateness) [1]. | **11** | List and define all variables for which data will be sought (such as PICO items, funding sources), any pre-planned data assumptions and simplifications. |
| **Risk of bias in individual studies:**  Qualitative and quantitative studies were analysed using the quality criteria of the National Institute for Health and Care Excellence (NICE) and mixed-methods studies were analysed using the “Mixed Methods Appraisal Tool (MMAT)”. | **12** | Describe methods used for assessing risk of bias of individual studies (including specification of whether this was done at the study or outcome level), and how this information is to be used in any data synthesis. |
| **Summary measures:**  Not applicable | **13** | State the principal summary measures (e.g., risk ratio, difference in means). |
| **Synthesis of results**  We did not perform a meta-analysis. A descriptive and qualitative analysis was performed [2]. | **14** | Describe the methods of handling data and combining results of studies, if done, including measures of consistency (e.g., I 2) for each meta-analysis. |
| **Risk of bias across studies:**  The critical appraisal performed showed that only three of the 28 publications (covering 26 studies) identified fulfilled all or most of the checklist criteria of NICE or MMAT. In accordance with the NICE grading system, the other publications fulfilled some (n=14) or a few (n=5) of the quality criteria and displayed a higher level of bias. | **15** | Specify any assessment of risk of bias that may affect the cumulative evidence (e.g., publication bias, selective reporting within studies). |
| **Additional analyses:**  Not applicable | **16** | Describe methods of additional analyses (e.g., sensitivity or subgroup analyses, meta-regression), if done, indicating which were pre-specified. |
| **RESULTS** | | |
| **Study selection:**  A flow diagram is provided in figure 1. | **17** | Give numbers of studies screened, assessed for eligibility, and included in the review, with reasons for exclusions at each stage, ideally with a flow diagram. |
| **Study characteristics**  The study characteristics are provided in Table 2. | **18** | For each study, present characteristics for which data were extracted (e.g., study size, PICOS, follow-up period) and provide the citations. |
| **Risk of bias within studies**  The risk of bias is provided in Table 2. | **19** | Present data on risk of bias of each study and, if available, any outcome level assessment (see item 12). |
| **Results of individual studies**  The results are provided in Table 2–4 and described in the manuscript. | **20** | For all outcomes considered (benefits or harms) present, for each study: (a) simple summary data for each intervention group (b) effect estimates and confidence intervals, ideally with a forest plot. |
| **Synthesis of results**  No meta-analysis was performed. | **21** | Present results of each meta-analysis done, including confidence intervals and measures of consistency. |
| **Risk of bias across studies**  The critical appraisal performed showed that only three of the 28 publications  (covering 26 studies) identified fulfilled all or most of the checklist criteria of  NICE or MMAT. In accordance with the NICE grading system, the other  publications fulfilled some (n = 14) or a few (n = 5) of the quality criteria and  displayed a higher level of bias (Table 2). | **22** | Present results of any assessment of risk of bias across studies (see Item 15). |
| **Additional analysis**  Not applicable | **23** | Give results of additional analyses, if done (e.g., sensitivity or subgroup analyses, meta-regression [see Item 16]). |
| **DISCUSSION** | | |
| **Summary of evidence**  To our knowledge, this is the first systematic review to analyse existing knowledge about information-seeking behaviours of people with DM. Wilson’s model proved to be most suitable during the systematic review analysis. Overall there were few studies with high heterogeneity regarding the research question, design, methods and participants. | **24** | Summarize the main findings including the strength of evidence for each main outcome; consider their relevance to key groups (e.g., healthcare providers, users, and policy makers). |
| **Limitations**  The inclusion criteria were handled rigorously, resulting in the inclusion of a small number of studies. A selection bias can be assumed because of the language and database restrictions. Studies published up to June 2015 were searched. In September 2016 and in June 2017, a forward citation tracking was performed in Google Scholar to find current relevant studies by searching studies that cited already-identified core papers. We also performed an update of two of our core databases (MEDLINE, CINAHL) and identified several new publications. However, it cannot be completely ruled out that some publications were missed after June 2015. | **25** | Discuss limitations at study and outcome level (e.g., risk of bias), and at review-level (e.g., incomplete retrieval of identified research, reporting bias). |
| **Conclusions**  There is a low number of studies analysing information-seeking behaviour, with a high heterogeneity regarding the research question, design, methods and participants. Both passive and active seeking seem to be performed by the patients; however, there may be a shift towards more-active information seeking behaviour. There is an association between information-seeking behaviour and demographics, socioeconomic and environmental aspects, source characteristics and individual needs, including variations due to the progression of the disease. Younger people with higher levels of education and higher incomes especially prefer to search for information on the Internet, which is, however, not a substitute for information provided by healthcare professionals. More well-performed studies are needed to re-evaluate existing models of patient information-seeking behaviour. | **26** | Provide a general interpretation of the results in the context of other evidence, and implications for future research. |
| **FUNDING** | | |
| **Funding**  The German Diabetes Study was initiated and is financed by the DDZ – German Diabetes Center, which is funded by the German Federal Ministry of Health (Berlin, Germany) and the Ministry of Innovation, Science, Research and Technology of the state North Rhine-Westphalia (Düsseldorf, Germany). The present analysis was funded by the German Federal Ministry of Education and Research (BMBF) within the German Center for Diabetes Research (DZD e.V.) and by the Research Commission of the Faculty of Medicine of the Heinrich Heine University Düsseldorf [9772577]. | **27** | Describe sources of funding for the systematic review and other support (e.g., supply of data); role of funders for the systematic review. |

**From: Moher D, Liberati A, Tetzlaff J, Altman DG, The PRISMA Group (2009). Preferred Reporting Items for Systematic Reviews and Meta-Analyses: The PRISMA Statement. PLoS Med 6(7): e1000097. doi:10.1371/journal.pmed1000097**

**References:**

1. Wilson TD. Human Information Behavior. Information Science. 2000:49–55.

2. Krippendorff K. Content analysis: An introduction to its methodology. 2nd ed. Thousand Oaks: Sage Publ; 2009.

3. National Institute for Health and Care Excellence. Methods for the development of NICE public health. 2012.

4.Pace R, Pluye P, Bartlett G, Macaulay AC, Salsberg J, Jagosh J, Seller R. Testing the reliability and efficiency of the pilot Mixed Methods Appraisal Tool (MMAT) for systematic mixed studies review. Int J Nurs Stud. 2012; doi:10.1016/j.ijnurstu.2009.01.009.

5. Pluye P, Gagnon M, Griffiths F, Johnson-Lafleur J. A scoring system for appraising mixed methods research, and concomitantly appraising qualitative, quantitative and mixed methods primary studies in Mixed Studies Reviews. Int J Nurs Stud. 2009; doi:10.1016/j.ijnurstu.2009.01.009.

6. Crowe M, Sheppard L. A general critical appraisal tool: an evaluation of construct validity. Int J Nurs Stud. 2011; doi:10.1016/j.ijnurstu.2011.06.004.
