# Supplementary material for: Diabetes-related information-seeking behaviour: a systematic review
Source: Syst Rev. 2017 Oct 24;6:212. doi: 10.1186/s13643-017-0602-8 (PMC5655894; doi:10.1186/s13643-017-0602-8)
Supplement: Supplementary file 2 — Update search strategy (MEDLINE, CINAHL) and search strategy (Ia and Ib). (DOC 343 kb) [file 13643_2017_602_MOESM2_ESM.doc]

**Appendix 2:**

**UPDATE, SEARCH STRATEGY (MEDLINE, CINAHL)**

#### MEDLINE (Pubmed)

Database: MEDLINE (1946–2017)

Research period: Entry date 02.07.2015–26.07.2017

Date of research: 26.07.2017

| **Step of search** | **Hits** | **Search terms** |
| --- | --- | --- |
|  |  | Filters: Publication date from 2015/07/02 |
| 1 | 287 | "information seeking behavior"[MeSH Terms] |
| 2 | 667 | "information literacy"[MeSH Terms] |
| 3 | 1,026 | "consumer health information"[MeSH Terms] |
| 4 | 3,110 | "patient education as topic"[MeSH Terms] |
| 5 | 249 | "health communication"[MeSH Terms] |
| 6 | 529 | 1 OR 5 |
| 7 | 16 | 6 AND (diabetes[Title/Abstract] OR diabetic[Title/Abstract] OR niddm[Title/Abstract] OR iddm[Title/Abstract] OR t2dm[Title/Abstract] OR t1dm[Title/Abstract] OR prediabetes[Title/Abstract] OR prediabetic[Title/Abstract] OR pre-diabetes[Title/Abstract] OR pre-diabetic[Title/Abstract] OR impaired glucose[Title/Abstract]) |
| 8 | 1,052 | 2 OR 3 |
| 9 | 24 | 8 AND (diabetes[Title/Abstract] OR diabetic[Title/Abstract] OR niddm [Title/Abstract] OR iddm[Title/Abstract] OR t2dm [Title/Abstract]OR t1dm[Title/Abstract] OR prediabetes[Title/Abstract] OR prediabetic[Title/Abstract] OR pre-diabetes[Title/Abstract] OR pre-diabetic[Title/Abstract] OR impaired glucose[Title/Abstract]) AND information[Title/Abstract] |
| 10 | 23 | 4 AND ((diabetes[Title/Abstract] OR diabetic[Title/Abstract] OR niddm[Title/Abstract] OR iddm[Title/Abstract] OR t2dm[Title/Abstract] OR t1dm[Title/Abstract] OR prediabetes[Title/Abstract] OR prediabetic[Title/Abstract] OR pre-diabetes[Title/Abstract] OR pre-diabetic[Title/Abstract] OR impaired glucose[Title/Abstract]) AND ((interest*[Title/Abstract] OR need[Title/Abstract] OR needs[Title/Abstract] OR question*[Title/Abstract] OR asking[Title/Abstract] OR ask[Title/Abstract] OR seek*[Title/Abstract] OR search*[Title/Abstract] OR demand[Title/Abstract] OR desire[Title/Abstract] OR request*[Title/Abstract] OR call[Title/Abstract] OR requirement[Title/Abstract] OR requiring[Title/Abstract] OR preference*[Title/Abstract] OR wish[Title/Abstract] OR wishes[Title/Abstract] OR provision*[Title/Abstract] OR expectation*[Title/Abstract]) AND information[Title/Abstract])) |
| 11 | 4 | (information needs[Title/Abstract]) AND (diabetes[Title/Abstract] OR diabetic[Title/Abstract] OR niddm[Title/Abstract] OR iddm[Title/Abstract] OR t2dm[Title/Abstract] OR t1dm[Title/Abstract] OR prediabetes[Title/Abstract] OR prediabetic[Title/Abstract] OR pre-diabetes[Title/Abstract] OR pre-diabetic[Title/Abstract] OR impaired glucose[Title/Abstract]) |
| 12 | 7,119 | (education[Title/Abstract] OR communication[Title/Abstract]) AND (need[Title/Abstract] OR needs[Title/Abstract] OR preference*[Title/Abstract]) |
| 13 | 128 | 12 AND (diabetes[Title] OR diabetic[Title] OR prediabetes[Title] OR prediabetic[Title] OR pre-diabetes[Title] OR pre-diabetic[Title] OR impaired glucose[Title]) |
| 14 | 1,885 | *PATIENT PREFERENCE/ |
| 15 | 20 | 14 AND (information[Title/Abstract] OR education[Title/Abstract] OR communication[Title/Abstract]) AND (diabetes[Title/Abstract] OR diabetic[Title/Abstract] OR prediabetes[Title/Abstract] OR prediabetic[Title/Abstract] OR pre-diabetes[Title/Abstract] OR pre-diabetic[Title/Abstract] OR impaired glucose[Title/Abstract]) |
| 16 | 19,229 | (patient[Title/Abstract] OR patient-centered[Title/Abstract]) AND (need[Title/Abstract] OR needs[Title/Abstract] OR seek*[Title/Abstract] OR search*[Title/Abstract] OR demand[Title/Abstract] OR desire[Title/Abstract] OR preference*[Title/Abstract] OR wish[Title/Abstract] OR wishes[Title/Abstract] OR provision*[Title/Abstract] OR expectation*Title/Abstract]) |
| 17 | 15 | 16 AND (diabetes[Title] OR diabetic[Title] OR prediabetes[Title] OR prediabetic[Title] OR pre-diabetes[Title] OR pre-diabetic[Title] OR impaired glucose[Title]) AND (need[Title] OR needs[Title] OR preference*[Title]) |
| 18 | 3 | 14 AND (facilitat*[Title] OR barrier*[Title] OR pitfall*[Title]) AND (diabetes [Title/Abstract] OR diabetic[Title/Abstract] OR prediabetes[Title/Abstract] OR prediabetic[Title/Abstract] OR pre-diabetes[Title/Abstract] OR pre-diabetic[Title/Abstract] OR impaired glucoseTitle/Abstract]) |
| 19 | 1,119 | (((information[Title/Abstract] OR education[Title/Abstract] OR communication[Title/Abstract]) AND (interest*[Title/Abstract] OR need[Title/Abstract] OR needs[Title/Abstract] OR question*[Title/Abstract] OR asking[Title/Abstract] OR ask[Title/Abstract] OR seek*[Title/Abstract] OR search*[Title/Abstract] OR demand[Title/Abstract] OR desire[Title/Abstract] OR request*[Title/Abstract] OR call[Title/Abstract] OR requirement[Title/Abstract] OR requiring[Title/Abstract] OR preference*[Title/Abstract] OR wish[Title/Abstract] OR wishes[Title/Abstract] OR provision*[Title/Abstract] OR expectation*[Title/Abstract])) AND (diabetes[Title/Abstract] OR diabetic[Title/Abstract] OR prediabetes[Title/Abstract] OR prediabetic[Title/Abstract] OR pre-diabetes[Title/Abstract] OR pre-diabetic[Title/Abstract] OR impaired glucose[Title/Abstract])) |
| 20 | 104 | 19 AND (interest*[Title] OR need*[Title] OR question*[Title] OR ask*[Title] OR talk*[Title] OR online communi*[Title] OR message*[Title] OR seek*[Title] OR search*[Title] OR demand[Title] OR desire[Title] OR request*[Title] OR call[Title] OR requir*[Title] OR preference*[Title] OR wish*[Title] OR perception*[Title] OR provision*[Title] OR expectation*[Title] OR facilitat*[Title] OR barrier*[Title] OR pitfall*[Title]) |
| 21 | 257 | information[Title] AND (interest*[Title] OR need[Title] OR needs[Title] OR question*[Title] OR asking[Title] OR ask[Title] OR seek*[Title] OR search*[Title] OR demand[Title] OR desire[Title] OR request*[Title] OR call[Title] OR requirement[Title] OR requiring[Title] OR preference*[Title]) |
| 22 | 5 | 21 AND (diabetes[Title/Abstract] OR diabetic[Title/Abstract] OR prediabetes[Title/Abstract] OR prediabetic[Title/Abstract] OR pre-diabetes[Title/Abstract] OR pre-diabetic[Title/Abstract] OR impaired glucose[Title/Abstract]) |
| 23 | 0 | identify*[Title] AND ((interest*[Title] OR need[Title] OR needs[Title] OR requirement[Title] OR preference*[Title]) AND (diabetes[Title] OR diabetic[Title] OR prediabetes[Title] OR prediabetic[Title] OR pre-diabetes[Title] OR pre-diabetic[Title] OR impaired glucose[Title]) |
| 24 | 7 | support*[Title] AND (interest*[Title] OR need[Title] OR needs[Title] OR requirement[Title] OR preference*Title]) AND (diabetes[Title/Abstract] OR diabetic[Title/Abstract] OR prediabetes[Title/Abstract] OR prediabetic[Title/Abstract] OR pre-diabetes[Title/Abstract] OR pre-diabetic[Title/Abstract] OR impaired glucose[Title/Abstract]) |
| 25 | 267 | 7 OR 9 OR 10 OR 11 OR 13 OR 15 OR 17 OR 18 OR 20 OR 22 OR 23 OR 24 |

#### CINAHL (EBSCO)

Database: CINAHL (1981-2015)

Research period: Entry date 01.08.2015-26.07.2017

Date of research: 31.08.2017

| **Step of search** | **Hits** | **Search terms** |
| --- | --- | --- |
| S1 | 235 | MW INFORMATION NEEDS AND (diabetes OR diabetic OR niddm OR iddm OR t2dm OR t1dm OR prediabetes OR prediabetic OR pre-diabetes OR pre-diabetic OR impaired glucose) |
| S2 | 17 | TI information needs AND (diabetes OR diabetic OR niddm OR iddm OR t2dm OR t1dm OR prediabetes OR prediabetic OR pre-diabetes OR pre-diabetic OR impaired glucose) |
| S3 | 67 | TI (education OR communication) AND TI (need OR needs OR preference*) AND (diabetes OR diabetic OR niddm OR iddm OR t2dm OR t1dm OR prediabetes OR prediabetic OR pre-diabetes OR pre-diabetic OR impaired glucose) |
| S4 | 181 | TI (patient OR patient-centered) AND TI (need* OR seek* OR talk* OR online communi* OR message OR search* OR demand OR desire OR preference* OR wish* OR provision* OR perception* OR expectation*) AND (diabetes OR diabetic OR niddm OR iddm OR t2dm OR t1dm OR prediabetes OR prediabetic OR pre-diabetes OR pre-diabetic OR impaired glucose) |
| S5 | 41 | TI information AND TI (interest* OR need OR needs OR question* OR asking OR ask OR seek* OR search* OR demand OR desire OR request* OR call OR requirement OR requiring OR preference*) AND (diabetes OR diabetic OR niddm OR iddm OR t2dm OR t1dm OR prediabetes OR prediabetic OR pre-diabetes OR pre-diabetic OR impaired glucose) |
| S6 | 494 | S1 OR S2 OR S3 OR S4 OR S5 |
| S7 | 23 | (EM 20150801-20170726) AND Exclude MEDLINE records AND S6 |

**SEARCH STRATEGY (Ib)**

#### 1.1 MEDLINE (OVID)

Database: MEDLINE (1946–2015)

Research period: Entry date 10.06.2014–01.07.2015

Date of research: 25.06.2015

| **Step of search** | **Hits** | **Search terms** |
| --- | --- | --- |
| 1 | 896 | EXP INFORMATION SEEKING BEHAVIOR/ |
| 2 | 2260 | EXP INFORMATION LITERACY/ |
| 3 | 4241 | EXP CONSUMER HEALTH INFORMATION/ |
| 4 | 72616 | EXP PATIENT EDUCATION AS TOPIC/ |
| 5 | 782 | EXP HEALTH COMMUNICATION/ |
| 6 | 1652 | 1 OR 5 |
| 7 | 35 | 6 AND (diabetes OR diabetic OR niddm OR iddm OR t2dm OR t1dm OR prediabetes OR prediabetic OR pre-diabetes OR pre-diabetic OR impaired glucose).ti,ab. |
| 8 | 4349 | 2 OR 3 |
| 9 | 87 | 8 AND (diabetes OR diabetic OR niddm OR iddm OR t2dm OR t1dm OR prediabetes OR prediabetic OR pre-diabetes OR pre-diabetic OR impaired glucose).ti,ab. AND information.ti,ab. |
| 10 | 108 | 4 AND ((diabetes OR diabetic OR niddm OR iddm OR t2dm OR t1dm OR prediabetes OR prediabetic OR pre-diabetes OR pre-diabetic OR impaired glucose).ti,ab. AND ((interest* OR need OR needs OR question* OR asking OR ask OR seek* OR search* OR demand OR desire OR request* OR call OR requirement OR requiring OR preference* OR wish OR wishes OR provision* OR expectation*) ADJ5 information)).ti,ab. |
| 11 | 47 | (information needs AND (diabetes OR diabetic OR niddm OR iddm OR t2dm OR t1dm OR prediabetes OR prediabetic OR pre-diabetes OR pre-diabetic OR impaired glucose)).ti,ab. |
| 12 | 4253 | ((education OR communication) ADJ2 (need OR needs OR preference*)).ti,ab. |
| 13 | 88 | 12 AND (diabetes OR diabetic OR prediabetes OR prediabetic OR pre-diabetes OR pre-diabetic OR impaired glucose).ti. |
| 14 | 2210 | *PATIENT PREFERENCE/ |
| 15 | 16 | 14 AND (information OR education OR communication).ti,ab. AND (diabetes OR diabetic OR prediabetes OR prediabetic OR pre-diabetes OR pre-diabetic OR impaired glucose).ti,ab. |
| 16 | 10638 | ((patient OR patient-centered) ADJ1 (need OR needs OR seek* OR search* OR demand OR desire OR preference* OR wish OR wishes OR provision* OR expectation*)).ti,ab. |
| 17 | 43 | 16 AND (diabetes OR diabetic OR prediabetes OR prediabetic OR pre-diabetes OR pre-diabetic OR impaired glucose).ti. AND (need OR needs OR preference*).ti. |
| 18 | 3 | 14 AND (facilitat* OR barrier* OR pitfall*).ti. AND (diabetes OR diabetic OR prediabetes OR prediabetic OR pre-diabetes OR pre-diabetic OR impaired glucose).ti,ab. |
| 19 | 1051 | (((information OR education OR communication) ADJ3 (interest* OR need OR needs OR question* OR asking OR ask OR seek* OR search* OR demand OR desire OR request* OR call OR requirement OR requiring OR preference* OR wish OR wishes OR provision* OR expectation*)) AND (diabetes OR diabetic OR prediabetes OR prediabetic OR pre-diabetes OR pre-diabetic OR impaired glucose)).ti,ab. |
| 20 | 194 | 19 AND (interest* OR need* OR question* OR ask* OR talk* OR online communi* OR message* OR seek* OR search* OR demand OR desire OR request* OR call OR requir* OR preference* OR wish* OR perception* OR provision* OR expectation* OR facilitat* OR barrier* OR pitfall*).ti. |
| 21 | 4256 | (information AND (interest* OR need OR needs OR question* OR asking OR ask OR seek* OR search* OR demand OR desire OR request* OR call OR requirement OR requiring OR preference*)).ti. |
| 22 | 57 | 21 AND (diabetes OR diabetic OR prediabetes OR prediabetic OR pre-diabetes OR pre-diabetic OR impaired glucose).ti,ab. |
| 23 | 5 | ((identify* ADJ2 (interest* OR need OR needs OR requirement OR preference*)) AND (diabetes OR diabetic OR prediabetes OR prediabetic OR pre-diabetes OR pre-diabetic OR impaired glucose)).ti. |
| 24 | 22 | (support* AND (interest* OR need OR needs OR requirement OR preference*)).ti. AND (diabetes OR diabetic OR prediabetes OR prediabetic OR pre-diabetes OR pre-diabetic OR impaired glucose).ti,ab. |
| 25 | 541 | 7 OR 9 OR 10 OR 11 OR 13 OR 15 OR 17 OR 18 OR 20 OR 22 OR 23 OR 24 |
| 26 | 50 | limit 25 to ed=‘20140610-20150701’ |

#### 1.2 EMBASE (OVID)

Database: EMBASE (1974–2015)

Research period: Entry date 24. KW 2014–27. KW 2015

Date of research: 25.06.2015

| **Step of search** | **Hits** | **Search terms** |
| --- | --- | --- |
| 1 | 1371 | EXP INFORMATION SEEKING/ |
| 2 | 238 | EXP INFORMATION LITERACY/ |
| 3 | 2647 | EXP CONSUMER HEALTH INFORMATION/ |
| 4 | 25038 | *PATIENT EDUCATION/ |
| 5 | 7879 | *MEDICAL INFORMATION/ |
| 6 | 102 | 1 AND 5 |
| 7 | 2 | 6 AND (diabetes OR diabetic OR prediabetes OR prediabetic OR pre-diabetes OR pre-diabetic OR impaired glucose).ti,ab. |
| 8 | 1595 | 1 OR 2 |
| 9 | 29 | 8 AND (diabetes OR diabetic OR prediabetes OR prediabetic OR pre-diabetes OR pre-diabetic OR impaired glucose).ti,ab. |
| 10 | 40 | 3 AND (diabetes OR diabetic OR prediabetes OR prediabetic OR pre-diabetes OR pre-diabetic OR impaired glucose).ti,ab. AND (interest* OR need OR needs OR question* OR asking OR ask OR seek* OR search* OR demand OR desire OR request* OR call OR requirement OR requiring OR preference* OR wish OR wishes OR provision* OR expectation*).ti,ab. |
| 11 | 69 | 5 AND (diabetes OR diabetic OR prediabetes OR prediabetic OR pre-diabetes OR pre-diabetic OR impaired glucose).ti,ab. AND (interest* OR need OR needs OR question* OR asking OR ask OR seek* OR search* OR demand OR desire OR request* OR call OR requirement OR requiring OR preference* OR wish OR wishes OR provision* OR expectation*).ti,ab. |
| 12 | 108 | 10 OR 11 |
| 13 | 25 | 12 AND ((information OR education OR support) ADJ2 (interest* OR need OR needs OR question* OR asking OR ask OR seek* OR search* OR demand OR desire OR request* OR call OR requirement OR requiring OR preference* OR wish OR wishes OR provision* OR expectation*)).ti,ab. |
| 14 | 44 | 4 AND ((diabetes OR diabetic OR niddm OR iddm OR t2dm OR t1dm OR prediabetes OR prediabetic OR pre-diabetes OR pre-diabetic OR impaired glucose).ti,ab. AND ((interest* OR need OR needs OR question* OR asking OR ask OR seek* OR search* OR demand OR desire OR request* OR call OR requirement OR requiring OR preference* OR wish OR wishes OR provision* OR expectation*) ADJ5 information)).ti,ab. |
| 15 | 69 | (information needs AND (diabetes OR diabetic OR niddm OR iddm OR t2dm OR t1dm OR prediabetes OR prediabetic OR pre-diabetes OR pre-diabetic OR impaired glucose)).ti,ab. |
| 16 | 5502 | ((education OR communication) ADJ2 (need OR needs OR preference*)).ti,ab. |
| 17 | 149 | 16 AND (diabetes OR diabetic OR prediabetes OR prediabetic OR pre-diabetes OR pre-diabetic OR impaired glucose).ti. |
| 18 | 1877 | *PATIENT PREFERENCE/ |
| 19 | 19 | 18 AND (information OR education OR communication).ti,ab. AND (diabetes OR diabetic OR prediabetes OR prediabetic OR pre-diabetes OR pre-diabetic OR impaired glucose).ti,ab. |
| 20 | 15113 | ((patient OR patient-centered) ADJ1 (need OR needs OR seek* OR search* OR demand OR desire OR preference* OR wish OR wishes OR provision* OR expectation*)).ti,ab. |
| 21 | 64 | 20 AND (diabetes OR diabetic OR prediabetes OR prediabetic OR pre-diabetes OR pre-diabetic OR impaired glucose).ti. AND (need OR needs OR preference*).ti. |
| 22 | 2 | 18 AND (facilitat* OR barrier* OR pitfall*).ti. AND (diabetes OR diabetic OR prediabetes OR prediabetic OR pre-diabetes OR pre-diabetic OR impaired glucose).ti,ab. |
| 23 | 5002 | (information AND (interest* OR need OR needs OR question* OR asking OR ask OR seek* OR search* OR demand OR desire OR request* OR call OR requirement OR requiring OR preference*)).ti. |
| 24 | 71 | 23 AND (diabetes OR diabetic OR prediabetes OR prediabetic OR pre-diabetes OR pre-diabetic OR impaired glucose).ti,ab. |
| 25 | 6 | ((identify* ADJ2 (interest* OR need OR needs OR requirement OR preference*)) AND (diabetes OR diabetic OR prediabetes OR prediabetic OR pre-diabetes OR pre-diabetic OR impaired glucose)).ti. |
| 26 | 42 | (support* AND (interest* OR need OR needs OR requirement OR preference*)).ti. AND (diabetes OR diabetic OR prediabetes OR prediabetic OR pre-diabetes OR pre-diabetic OR impaired glucose).ti,ab. |
| 27 | 984 | (((information OR education OR communication) ADJ2 (interest* OR need OR needs OR question* OR asking OR ask OR seek* OR search* OR demand OR desire OR request* OR call OR requirement OR requiring OR preference* OR wish OR wishes OR provision* OR expectation*)) AND (diabetes OR diabetic OR prediabetes OR prediabetic OR pre-diabetes OR pre-diabetic OR impaired glucose)).ti,ab. |
| 28 | 175 | 27 AND (interest* OR need* OR question* OR ask* OR talk* OR online communi* OR message* OR seek* OR search* OR demand OR desire OR request* OR call OR requir* OR preference* OR wish* OR perception* OR provision* OR expectation* OR facilitat* OR barrier* OR pitfall*).ti. |
| 29 | 510 | 7 OR 9 OR 13 OR 14 OR 15 OR 17 OR 19 OR 21 OR 22 OR 24 OR 25 OR 26 OR 28 |
| 30 | 67 | limit 29 to em=‘201424-201527’ |

**1.3 PsycINFO, Journals@OVID (OVID)**

Database: PsycINFO (1806-2015)

Journals@OVID

Research period: Update 10.06.2014–01.07.2015 (PsycInfo) and publication years

2014–2015 (Journals@Ovid)

Date of research: 25.06.2015

| **Step of search** | **Hits** | **Search terms** |
| --- | --- | --- |
| 1 | 3175 | EXP INFORMATION SEEKING/ |
| 2 | 19 | 1 AND (diabetes OR diabetic OR prediabetes OR prediabetic OR pre-diabetes OR pre-diabetic OR impaired glucose).ti,ab. |
| 3 | 3209 | CLIENT EDUCATION/ |
| 4 | 10 | 3 AND (diabetes OR diabetic OR prediabetes OR prediabetic OR pre-diabetes OR pre-diabetic OR impaired glucose).ti,ab. AND ((interest* OR need OR needs OR question* OR asking OR ask OR seek* OR search* OR demand OR desire OR request* OR call OR requirement OR requiring OR preference* OR wish OR wishes OR provision* OR expectation*) ADJ5 information).ti,ab. |
| 5 | 30 | (information needs AND (diabetes OR diabetic OR niddm OR iddm OR t2dm OR t1dm OR prediabetes OR prediabetic OR pre-diabetes OR pre-diabetic OR impaired glucose)).ti,ab. |
| 6 | 6616 | ((education OR communication) ADJ2 (need OR needs OR preference*)).ti,ab. |
| 7 | 100 | 6 AND (diabetes OR diabetic OR prediabetes OR prediabetic OR pre-diabetes OR pre-diabetic OR impaired glucose).ti. |
| 8 | 8087 | ((patient OR patient-centered) ADJ1 (need OR needs OR seek* OR search* OR demand OR desire OR preference* OR wish OR wishes OR provision* OR expectation*)).ti,ab. |
| 9 | 29 | 8 AND (diabetes OR diabetic OR prediabetes OR prediabetic OR pre-diabetes OR pre-diabetic OR impaired glucose).ti. AND (need OR needs OR preference*).ti. |
| 10 | 4835 | (information AND (interest* OR need OR needs OR question* OR asking OR ask OR seek* OR search* OR demand OR desire OR request* OR call OR requirement OR requiring OR preference*)).ti. |
| 11 | 35 | 10 AND (diabetes OR diabetic OR prediabetes OR prediabetic OR pre-diabetes OR pre-diabetic OR impaired glucose).ti,ab. |
| 12 | 9 | ((identify* ADJ2 (interest* OR need OR needs OR requirement OR preference*)) AND (diabetes OR diabetic OR prediabetes OR prediabetic OR pre-diabetes OR pre-diabetic OR impaired glucose)).ti. |
| 13 | 38 | (support* AND (interest* OR need OR needs OR requirement OR preference*)).ti. AND (diabetes OR diabetic OR prediabetes OR prediabetic OR pre-diabetes OR pre-diabetic OR impaired glucose).ti,ab. |
| 14 | 572 | (((information OR education OR communication) ADJ2 (interest* OR need OR needs OR question* OR asking OR ask OR seek* OR search* OR demand OR desire OR request* OR call OR requirement OR requiring OR preference* OR wish OR wishes OR provision* OR expectation*)) AND (diabetes OR diabetic OR prediabetes OR prediabetic OR pre-diabetes OR pre-diabetic OR impaired glucose)).ti,ab. |
| 15 | 136 | 14 AND (interest* OR need* OR question* OR ask* OR talk* OR online communi* OR message* OR seek* OR search* OR demand OR desire OR request* OR call OR requir* OR preference* OR wish* OR perception* OR provision* OR expectation* OR facilitat* OR barrier* OR pitfall*).ti. |
| 16 | 294 | 2 OR 4 OR 5 OR 7 OR 9 OR 11 OR 12 OR 13 OR 15 |
| 17 | 210 | limit 16 to up=‘20140610-20150701’ |
| 18 | 209 | remove duplicates from 17 |
| 19 | 57 | limit 18 to yr=‘2013 - 2015’  Journals@Ovid: 43  PsycInfo: 14 |

#### 1.4 CINAHL (EBSCO)

Database: CINAHL (1981–2015)

Research period: Entry date 01.06.2014–31.07.2015

Date of research: 26.06.2015

| **Step of search** | **Hits** | **Search terms** |
| --- | --- | --- |
| S1 | 206 | MW INFORMATION NEEDS AND (diabetes OR diabetic OR niddm OR iddm OR t2dm OR t1dm OR prediabetes OR prediabetic OR pre-diabetes OR pre-diabetic OR impaired glucose) |
| S2 | 7 | TI information needs AND (diabetes OR diabetic OR niddm OR iddm OR t2dm OR t1dm OR prediabetes OR prediabetic OR pre-diabetes OR pre-diabetic OR impaired glucose) |
| S3 | 51 | TI (education OR communication) AND TI (need OR needs OR preference*) AND (diabetes OR diabetic OR niddm OR iddm OR t2dm OR t1dm OR prediabetes OR prediabetic OR pre-diabetes OR pre-diabetic OR impaired glucose) |
| S4 | 110 | TI (patient OR patient-centered) AND TI (need* OR seek* OR talk* OR online communi* OR message OR search* OR demand OR desire OR preference* OR wish* OR provision* OR perception* OR expectation*) AND (diabetes OR diabetic OR niddm OR iddm OR t2dm OR t1dm OR prediabetes OR prediabetic OR pre-diabetes OR pre-diabetic OR impaired glucose) |
| S5 | 22 | TI information AND TI (interest* OR need OR needs OR question* OR asking OR ask OR seek* OR search* OR demand OR desire OR request* OR call OR requirement OR requiring OR preference*) AND (diabetes OR diabetic OR niddm OR iddm OR t2dm OR t1dm OR prediabetes OR prediabetic OR pre-diabetes OR pre-diabetic OR impaired glucose) |
| S6 | 372 | S1 OR S2 OR S3 OR S4 OR S5 |
| S7 | 21 | (EM 20140601-20150731) AND S6 |

#### 1.5 The Cochrane Library (Wiley)

Databases: Cochrane Database of Systematic Reviews (1996–2015)

Database of Abstracts of Reviews of Effects (DARE) (1994–2015)

Cochrane Central Register of Controlled Trials (1898–2015)

Cochrane Methodology Register (1904–2012)

Health Technology Assessment (HTA) (1989–2015)

NHS Economic Evaluation Database (NHS EED) (1968–2015)

Research period: Date of publication 06.2014–06.2015

Date of research: 26.06.2015

| **Step of search** | **Hits** | **Search terms** |
| --- | --- | --- |
| #1 | 0 | (information need* AND (diabetes OR diabetic OR prediabetes OR prediabetic OR pre-diabetes OR pre-diabetic OR impaired glucose)):ti |
| #2 | 4 | ((education OR communication OR information) AND (need OR needs OR preference*) AND (diabetes OR diabetic OR niddm OR iddm OR t2dm OR t1dm OR prediabetes OR prediabetic OR pre-diabetes OR pre-diabetic OR impaired glucose)):ti |
| #3 | 8 | ((information* OR knowledge) AND (need OR needs OR needed OR seek* OR talk* OR online communi* OR search* OR demand OR desire OR preference* OR wish* OR provision* OR perception* OR expectation*) AND (diabetes OR diabetic OR niddm OR iddm OR t2dm OR t1dm OR prediabetes OR prediabetic OR pre-diabetes OR pre-diabetic OR impaired glucose)):ti |
| #4 | 3 | (information AND (interest* OR need OR needs OR question* OR asking OR ask OR seek* OR search* OR demand OR desire OR request* OR call OR requirement OR requiring OR preference*) AND (diabetes OR diabetic OR niddm OR iddm OR t2dm OR t1dm OR prediabetes OR prediabetic OR pre-diabetes OR pre-diabetic OR impaired glucose)):ti |
| #5 | 40 | ((need OR needs OR seek* OR search* OR demand OR desire OR preference* OR wish OR wishes OR provision* OR expectation*) AND (diabetes OR diabetic OR prediabetes OR prediabetic OR pre-diabetes OR pre-diabetic OR impaired glucose) AND (information OR education OR support)):ti |
| #6 | 117 | (information need AND (diabetes OR diabetic OR prediabetes OR prediabetic OR pre-diabetes OR pre-diabetic OR impaired glucose)):ti,ab,kw |
| #7 | 7 | #6 AND (information OR knowledge):ti |
| #5 | 46 | #1 OR #2 OR #3 OR #4 OR #5 OR #7  Cochrane Reviews: 1  Other Reviews: 20  Trials: 10  Methods Studies: 2  Technology Assessments: 0  Economic Evaluations: 13  Cochrane Groups: 0 |
| #6 | 0 | #1 OR #2 OR #3 OR #4 OR #5 OR #7 Online Publication Date in the last 12 months |

#### 1.6 Web of Science (Thomson Reuters)

Database: Web of Science (1950–2015)

Research period: Publication years 2014–2015

Date of research: 26.06.2015

| **Step of search** | **Hits** | **Search terms** |
| --- | --- | --- |
| 1 | 163 | TITLE: (information AND (interest* OR need OR needs OR question* OR talk* OR ask* OR online communi* OR seek* OR search* OR demand OR desire OR request* OR call OR requir* OR perception* OR preference*) AND (diabetes OR diabetic OR prediabetes OR prediabetic OR pre-diabetes OR pre-diabetic OR impaired glucose)) OR  TITLE: ((need OR needs OR seek* OR search* OR demand OR desire OR preference* OR wish OR wishes OR provision* OR expectation*) AND (diabetes OR diabetic OR prediabetes OR prediabetic OR pre-diabetes OR pre-diabetic OR impaired glucose) AND (information OR education OR support OR perception*)) OR  TITLE: ((education OR communication) AND (need OR needs OR preference*) AND (diabetes OR diabetic OR prediabetes OR prediabetic OR pre-diabetes OR pre-diabetic OR impaired glucose))  Timespan=All Years  Lemmatization=On |
| 2 | 26 | TITLE: ((information AND (interest* OR need OR needs OR question* OR talk* OR ask* OR online communi* OR seek* OR search* OR demand OR desire OR request* OR call OR requir* OR perception* OR preference*) AND (diabetes OR diabetic OR prediabetes OR prediabetic OR pre-diabetes OR pre-diabetic OR impaired glucose))) OR TITLE: (((need OR needs OR seek* OR search* OR demand OR desire OR preference* OR wish OR wishes OR provision* OR expectation*) AND (diabetes OR diabetic OR prediabetes OR prediabetic OR pre-diabetes OR pre-diabetic OR impaired glucose) AND (information OR education OR support OR perception*))) OR TITLE: (((education OR communication) AND (need OR needs OR preference*) AND (diabetes OR diabetic OR prediabetes OR prediabetic OR pre-diabetes OR pre-diabetic OR impaired glucose)))  Refined by: PUBLICATION YEARS: (2015 OR 2014)  Timespan=All years  Search language=Auto |

#### 1.7 ERIC (Institute of Education Sciences)

Database: ERIC (1966–2015)

Research period: unlimited

Date of research: 26.06.2015

| **Step of search** | **Hits** | **Search terms** |
| --- | --- | --- |
| 1 | 7 | ‘INFORMATION NEED*’ AND ‘DIABETES’ |

#### 1.8 ScienceDirect (Elsevier)

Database: ScienceDirect (Elsevier, Segmente ‘Decision Sciences, Medicine’, ‘Medicine and Dentistry’, ‘Neuroscience’, ‘Nursing and Health professions’, ‘Pharmacology, Toxicology and Pharmaceutical Science’,

‘Psychology’,‘Social Sciences”) ‘1823–2015)

Research period: Publication years 2014–2015

Date of research: 26.06.2015

| **Step of search** | **Hits** | **Search terms** |
| --- | --- | --- |
| 1 | 11 | TITLE((information) AND (diabetes OR diabetic)) AND TITLE-ABSTR-KEY((interest* OR need OR needs OR question* OR ask* OR talk* OR online communi* OR seek* OR search* OR demand OR desire OR request* OR call OR requir* OR perception* OR preference*)) |
| 2 | 4 | pub-date > 2013 and TITLE((information) AND (diabetes OR diabetic)) AND TITLE-ABSTR-KEY((interest* OR need OR needs OR question* OR ask* OR talk* OR online communi* OR seek* OR search* OR demand OR desire OR request* OR call OR requir* OR perception* OR preference*)) |

#### 1.9 CCMed (MedPilot, ZBMed)

Database: CCMed (2001–2015)

Research period: Publication years 2014–2015

Date of research: 26.06.2015

| **Step of search** | **Hits** | **Search terms** |
| --- | --- | --- |
| 1 | 37 | TI=((diabetes OR diabetic OR pre-diabet* OR prediabet* OR impaired glucose) AND (interest* OR need* OR question* OR talk* OR online communi* OR ask* OR seek* OR search* OR demand OR desire OR request* OR call OR requir* OR perception* OR preference*)) AND TI=((information* OR education OR knowledge))  Jahre 2014-2015: 1 |
| 2 | 392 | TI=((diabetes OR diabetic OR pre-diabet* OR prediabet* OR impaired glucose)) AND TI=((information* OR education OR knowledge))  Jahre 2014-2015: 21 |

**1.10 *Deutsches Ärzteblatt* (Deutscher Ärzteverlag)**

Database: Deutsches Ärzteblatt (1996–2015)

Research period: Publication years 2014–2015

Date of research: 26.06.2015

| **Step of search** | **Hits** | **Search terms** |
| --- | --- | --- |
| 1 | 0 | Diabetes informationsbedarf  Publikationsjahre 2014–2015 |
| 2 | 0 | Diabetes information need  Publikationsjahre 2014–2015 |

#### 1.11 Karlsruher virtueller Katalog (KvK)(Karlsruher Institut für Technologie)

Database: Karlsruher virtueller Katalog (DIMDI)

Research period: unlimited

Date of research: 26.06.2015

| **Step of search** | **Hits^1^** | **Search terms** |
| --- | --- | --- |
| 1 | 0 | information need* AND (diabet* OR pre-diabet* OR prediabet*) |
| 2 | 0 | informationsbedarf AND (diabet* OR pre-diabet* OR prediabet*) |
| 3 | 0 | preferences AND (diabet* OR pre-diabet* OR prediabet*) |

^1^after Screening

**SEARCH STRATEGY (Ia)**

1. *MEDLINE (OVID)*

Database: MEDLINE (1946-2014)

Research period: unlimited

| **Search step** | **Hits** | **Search** |
| --- | --- | --- |
| 1 | 702 | EXP INFORMATION SEEKING BEHAVIOR/ |
| 2 | 1718 | EXP INFORMATION LITERACY/ |
| 3 | 3397 | EXP CONSUMER HEALTH INFORMATION/ |
| 4 | 70372 | EXP PATIENT EDUCATION AS TOPIC/ |
| 5 | 600 | EXP HEALTH COMMUNICATION/ |
| 6 | 1279 | 1 OR 5 |
| 7 | 34 | 6 AND (diabetes OR diabetic OR niddm OR iddm OR t2dm OR t1dm OR prediabetes OR prediabetic OR pre-diabetes OR pre-diabetic OR impaired glucose).ti,ab. |
| 8 | 3488 | 2 OR 3 |
| 9 | 80 | 8 AND (diabetes OR diabetic OR niddm OR iddm OR t2dm OR t1dm OR prediabetes OR prediabetic OR pre-diabetes OR pre-diabetic OR impaired glucose).ti,ab. AND information.ti,ab. |
| 10 | 107 | 4 AND ((diabetes OR diabetic OR niddm OR iddm OR t2dm OR t1dm OR prediabetes OR prediabetic OR pre-diabetes OR pre-diabetic OR impaired glucose).ti,ab. AND ((interest* OR need OR needs OR question* OR asking OR ask OR seek* OR search* OR demand OR desire OR request* OR call OR requirement OR requiring OR preference* OR wish OR wishes OR provision* OR expectation*) ADJ5 information)).ti,ab. |
| 11 | 45 | (information needs AND (diabetes OR diabetic OR niddm OR iddm OR t2dm OR t1dm OR prediabetes OR prediabetic OR pre-diabetes OR pre-diabetic OR impaired glucose)).ti,ab. |
| 12 | 3935 | ((education OR communication) ADJ2 need OR needs OR preference*)).ti,ab. |
| 13 | 85 | 12 AND (diabetes OR diabetic OR prediabetes OR prediabetic OR pre-diabetes OR pre-diabetic OR impaired glucose).ti. |
| 14 | 1721 | *PATIENT PREFERENCE/ |
| 15 | 13 | 14 AND (information OR education OR communication).ti,ab. AND (diabetes OR diabetic OR prediabetes OR prediabetic OR pre-diabetes OR pre-diabetic OR impaired glucose).ti,ab. |
| 16 | 9730 | ((patient OR patient-centered) ADJ1 (need OR needs OR seek* OR search* OR demand OR desire OR preference* OR wish OR wishes OR provision* OR expectation*)).ti,ab. |
| 17 | 34 | 16 AND (diabetes OR diabetic OR prediabetes OR prediabetic OR pre-diabetes OR pre-diabetic OR impaired glucose).ti. AND (need OR needs OR preference*).ti. |
| 18 | 2 | 14 AND (facilitat* OR barrier* OR pitfall*).ti. AND (diabetes OR diabetic OR prediabetes OR prediabetic OR pre-diabetes OR pre-diabetic OR impaired glucose).ti,ab. |
| 19 | 941 | (((information OR education OR communication) ADJ3 (interest* OR need OR needs OR question* OR asking OR ask OR seek* OR search* OR demand OR desire OR request* OR call OR requirement OR requiring OR preference* OR wish OR wishes OR provision* OR expectation*)) AND (diabetes OR diabetic OR prediabetes OR prediabetic OR pre-diabetes OR pre-diabetic OR impaired glucose)).ti,ab. |
| 20 | 178 | 19 AND (interest* OR need* OR question* OR ask* OR talk* OR online communi* OR message* OR seek* OR search* OR demand OR desire OR request* OR call OR requir* OR preference* OR wish* OR perception* OR provision* OR expectation* OR facilitat* OR barrier* OR pitfall*).ti. |
| 21 | 4014 | (information AND (interest* OR need OR needs OR question* OR asking OR ask OR seek* OR search* OR demand OR desire OR request* OR call OR requirement OR requiring OR preference*)).ti. |
| 22 | 53 | 21 AND (diabetes OR diabetic OR prediabetes OR prediabetic OR pre-diabetes OR pre-diabetic OR impaired glucose).ti,ab. |
| 23 | 5 | ((identify* ADJ2 (interest* OR need OR needs OR requirement OR preference*)) AND (diabetes OR diabetic OR prediabetes OR prediabetic OR pre-diabetes OR pre-diabetic OR impaired glucose)).ti. |
| 24 | 19 | (support* AND (interest* OR need OR needs OR requirement OR preference*)).ti. AND (diabetes OR diabetic OR prediabetes OR prediabetic OR pre-diabetes OR pre-diabetic OR impaired glucose).ti,ab. |
| 25 | 497 | 7 OR 9 OR 10 OR 11 OR 13 OR 15 OR 17 OR 18 OR 20 OR 22 OR 23 OR 24 |

1. *EMBASE (OVID)*

Database: EMBASE (1974-2014)

Research period: unlimited

| Search step | Hits | Search |
| --- | --- | --- |
| 1 | 989 | EXP INFORMATION SEEKING/ |
| 2 | 187 | EXP INFORMATION LITERACY/ |
| 3 | 2226 | EXP CONSUMER HEALTH INFORMATION/ |
| 4 | 23953 | *PATIENT EDUCATION/ |
| 5 | 7326 | *MEDICAL INFORMATION/ |
| 6 | 72 | 1 AND 5 |
| 7 | 2 | 6 AND (diabetes OR diabetic OR prediabetes OR prediabetic OR pre-diabetes OR pre-diabetic OR impaired glucose).ti,ab. |
| 8 | 1164 | 1 OR 2 |
| 9 | 20 | 8 AND (diabetes OR diabetic OR prediabetes OR prediabetic OR pre-diabetes OR pre-diabetic OR impaired glucose).ti,ab. |
| 10 | 36 | 3 AND (diabetes OR diabetic OR prediabetes OR prediabetic OR pre-diabetes OR pre-diabetic OR impaired glucose).ti,ab. AND (interest* OR need OR needs OR question* OR asking OR ask OR seek* OR search* OR demand OR desire OR request* OR call OR requirement OR requiring OR preference* OR wish OR wishes OR provision* OR expectation*).ti,ab. |
| 11 | 65 | 5 AND (diabetes OR diabetic OR prediabetes OR prediabetic OR pre-diabetes OR pre-diabetic OR impaired glucose).ti,ab. AND (interest* OR need OR needs OR question* OR asking OR ask OR seek* OR search* OR demand OR desire OR request* OR call OR requirement OR requiring OR preference* OR wish OR wishes OR provision* OR expectation*).ti,ab. |
| 12 | 100 | 10 OR 11 |
| 13 | 24 | 12 AND ((information OR education OR support) ADJ2 (interest* OR need OR needs OR question* OR asking OR ask OR seek* OR search* OR demand OR desire OR request* OR call OR requirement OR requiring OR preference* OR wish OR wishes OR provision* OR expectation*)).ti,ab. |
| 14 | 43 | 4 AND ((diabetes OR diabetic OR niddm OR iddm OR t2dm OR t1dm OR prediabetes OR prediabetic OR pre-diabetes OR pre-diabetic OR impaired glucose).ti,ab. AND ((interest* OR need OR needs OR question* OR asking OR ask OR seek* OR search* OR demand OR desire OR request* OR call OR requirement OR requiring OR preference* OR wish OR wishes OR provision* OR expectation*) ADJ5 information)).ti,ab. |
| 15 | 65 | (information needs AND (diabetes OR diabetic OR niddm OR iddm OR t2dm OR t1dm OR prediabetes OR prediabetic OR pre-diabetes OR pre-diabetic OR impaired glucose)).ti,ab. |
| 16 | 4941 | ((education OR communication) ADJ2 (need OR needs OR preference*)).ti,ab. |
| 17 | 127 | 16 AND (diabetes OR diabetic OR prediabetes OR prediabetic OR pre-diabetes OR pre-diabetic OR impaired glucose).ti. |
| 18 | 1354 | *PATIENT PREFERENCE/ |
| 19 | 13 | 18 AND (information OR education OR communication).ti,ab. AND (diabetes OR diabetic OR prediabetes OR prediabetic OR pre-diabetes OR pre-diabetic OR impaired glucose).ti,ab. |
| 20 | 13294 | ((patient OR patient-centered) ADJ1 (need OR needs OR seek* OR search* OR demand OR desire OR preference* OR wish OR wishes OR provision* OR expectation*)).ti,ab. |
| 21 | 52 | 20 AND (diabetes OR diabetic OR prediabetes OR prediabetic OR pre-diabetes OR pre-diabetic OR impaired glucose).ti. AND (need OR needs OR preference*).ti. |
| 22 | 2 | 18 AND (facilitat* OR barrier* OR pitfall*).ti. AND (diabetes OR diabetic OR prediabetes OR prediabetic OR pre-diabetes OR pre-diabetic OR impaired glucose).ti,ab. |
| 23 | 4637 | (information AND (interest* OR need OR needs OR question* OR asking OR ask OR seek* OR search* OR demand OR desire OR request* OR call OR requirement OR requiring OR preference*)).ti. |
| 24 | 66 | 23 AND (diabetes OR diabetic OR prediabetes OR prediabetic OR pre-diabetes OR pre-diabetic OR impaired glucose).ti,ab. |
| 25 | 6 | ((identify* ADJ2 (interest* OR need OR needs OR requirement OR preference*)) AND (diabetes OR diabetic OR prediabetes OR prediabetic OR pre-diabetes OR pre-diabetic OR impaired glucose)).ti. |
| 26 | 32 | (support* AND (interest* OR need OR needs OR requirement OR preference*)).ti. AND (diabetes OR diabetic OR prediabetes OR prediabetic OR pre-diabetes OR pre-diabetic OR impaired glucose).ti,ab. |
| 27 | 855 | (((information OR education OR communication) ADJ2 (interest* OR need OR needs OR question* OR asking OR ask OR seek* OR search* OR demand OR desire OR request* OR call OR requirement OR requiring OR preference* OR wish OR wishes OR provision* OR expectation*)) AND (diabetes OR diabetic OR prediabetes OR prediabetic OR pre-diabetes OR pre-diabetic OR impaired glucose)).ti,ab. |
| 28 | 158 | 27 AND (interest* OR need* OR question* OR ask* OR talk* OR online communi* OR message* OR seek* OR search* OR demand OR desire OR request* OR call OR requir* OR preference* OR wish* OR perception* OR provision* OR expectation* OR facilitat* OR barrier* OR pitfall*).ti. |
| 29 | 440 | 7 OR 9 OR 13 OR 14 OR 15 OR 17 OR 19 OR 21 OR 22 OR 24 OR 25 OR 26 OR 28 |

1. *PsycINFO, Journals@OVID (OVID)*

Databases: PsycINFO (1806-2014), Journals@OVID

Research period: unlimited

| **Search step** | **Hits** | **Search** |
| --- | --- | --- |
| 1 | 3002 | EXP INFORMATION SEEKING/ |
| 2 | 15 | 1 AND (diabetes OR diabetic OR prediabetes OR prediabetic OR pre-diabetes OR pre-diabetic OR impaired glucose).ti,ab. |
| 3 | 3027 | CLIENT EDUCATION/ |
| 4 | 7 | 3 AND (diabetes OR diabetic OR prediabetes OR prediabetic OR pre-diabetes OR pre-diabetic OR impaired glucose).ti,ab. AND ((interest* OR need OR needs OR question* OR asking OR ask OR seek* OR search* OR demand OR desire OR request* OR call OR requirement OR requiring OR preference* OR wish OR wishes OR provision* OR expectation*) ADJ5 information).ti,ab. |
| 5 | 28 | (information needs AND (diabetes OR diabetic OR niddm OR iddm OR t2dm OR t1dm OR prediabetes OR prediabetic OR pre-diabetes OR pre-diabetic OR impaired glucose)).ti,ab. |
| 6 | 5992 | ((education OR communication) ADJ2 (need OR needs OR preference*)).ti,ab. |
| 7 | 91 | 6 AND (diabetes OR diabetic OR prediabetes OR prediabetic OR pre-diabetes OR pre-diabetic OR impaired glucose).ti. |
| 8 | 7387 | ((patient OR patient-centered) ADJ1 (need OR needs OR seek* OR search* OR demand OR desire OR preference* OR wish OR wishes OR provision* OR expectation*)).ti,ab. |
| 9 | 26 | 8 AND (diabetes OR diabetic OR prediabetes OR prediabetic OR pre-diabetes OR pre-diabetic OR impaired glucose).ti. AND (need OR needs OR preference*).ti. |
| 10 | 4494 | (information AND (interest* OR need OR needs OR question* OR asking OR ask OR seek* OR search* OR demand OR desire OR request* OR call OR requirement OR requiring OR preference*)).ti. |
| 11 | 30 | 10 AND (diabetes OR diabetic OR prediabetes OR prediabetic OR pre-diabetes OR pre-diabetic OR impaired glucose).ti,ab. |
| 12 | 9 | ((identify* ADJ2 (interest* OR need OR needs OR requirement OR preference*)) AND (diabetes OR diabetic OR prediabetes OR prediabetic OR pre-diabetes OR pre-diabetic OR impaired glucose)).ti. |
| 13 | 29 | (support* AND (interest* OR need OR needs OR requirement OR preference*)).ti. AND (diabetes OR diabetic OR prediabetes OR prediabetic OR pre-diabetes OR pre-diabetic OR impaired glucose).ti,ab. |
| 14 | 509 | (((information OR education OR communication) ADJ2 (interest* OR need OR needs OR question* OR asking OR ask OR seek* OR search* OR demand OR desire OR request* OR call OR requirement OR requiring OR preference* OR wish OR wishes OR provision* OR expectation*)) AND (diabetes OR diabetic OR prediabetes OR prediabetic OR pre-diabetes OR pre-diabetic OR impaired glucose)).ti,ab. |
| 15 | 123 | 14 AND (interest* OR need* OR question* OR ask* OR talk* OR online communi* OR message* OR seek* OR search* OR demand OR desire OR request* OR call OR requir* OR preference* OR wish* OR perception* OR provision* OR expectation* OR facilitat* OR barrier* OR pitfall*).ti. |
| 16 | 259 | 2 OR 4 OR 5 OR 7 OR 9 OR 11 OR 12 OR 13 OR 15 |

1. *CINAHL (EBSCO)*

Database: CINAHL (1981-2014)

Research period: unlimited

| **Search step** | **Hits** | **Search** |
| --- | --- | --- |
| S1 | 198 | MW INFORMATION NEEDS AND (diabetes OR diabetic OR niddm OR iddm OR t2dm OR t1dm OR prediabetes OR prediabetic OR pre-diabetes OR pre-diabetic OR impaired glucose) |
| S2 | 8 | TI information needs AND (diabetes OR diabetic OR niddm OR iddm OR t2dm OR t1dm OR prediabetes OR prediabetic OR pre-diabetes OR pre-diabetic OR impaired glucose) |
| S3 | 57 | TI (education OR communication) AND TI (need OR needs OR preference*) AND (diabetes OR diabetic OR niddm OR iddm OR t2dm OR t1dm OR prediabetes OR prediabetic OR pre-diabetes OR pre-diabetic OR impaired glucose) |
| S4 | 103 | TI (patient OR patient-centered) AND TI (need* OR seek* OR talk* OR online communi* OR message OR search* OR demand OR desire OR preference* OR wish* OR provision* OR perception* OR expectation*) AND (diabetes OR diabetic OR niddm OR iddm OR t2dm OR t1dm OR prediabetes OR prediabetic OR pre-diabetes OR pre-diabetic OR impaired glucose) |
| S5 | 20 | TI information AND TI (interest* OR need OR needs OR question* OR asking OR ask OR seek* OR search* OR demand OR desire OR request* OR call OR requirement OR requiring OR preference*) AND (diabetes OR diabetic OR niddm OR iddm OR t2dm OR t1dm OR prediabetes OR prediabetic OR pre-diabetes OR pre-diabetic OR impaired glucose) |
| S6 | 352 | S1 OR S2 OR S3 OR S4 OR S5 |

1. *The Cochrane Library (Wiley)*

Databases: Cochrane Database of Systematic Reviews (1996-2014)

Database of Abstracts of Reviews of Effects (DARE) (1994-2014)

Cochrane Central Register of Controlled Trials (1898-2014)

Cochrane Methodology Register (1904-2012)

Health Technology Assessment (HTA) (1989-2014)

NHS Economic Evaluation Database (NHS EED) (1968-2014)

Research period: unlimited

| **Search step** | **Hits** | **Search** |
| --- | --- | --- |
| #1 | 0 | (information need* AND (diabetes OR diabetic OR prediabetes OR prediabetic OR pre-diabetes OR pre-diabetic OR impaired glucose)):ti |
| #2 | 4 | ((education OR communication OR information) AND (need OR needs OR preference*) AND (diabetes OR diabetic OR niddm OR iddm OR t2dm OR t1dm OR prediabetes OR prediabetic OR pre-diabetes OR pre-diabetic OR impaired glucose)):ti |
| #3 | 8 | ((information* OR knowledge) AND (need OR needs OR needed OR seek* OR talk* OR online communi* OR search* OR demand OR desire OR preference* OR wish* OR provision* OR perception* OR expectation*) AND (diabetes OR diabetic OR niddm OR iddm OR t2dm OR t1dm OR prediabetes OR prediabetic OR pre-diabetes OR pre-diabetic OR impaired glucose)):ti |
| #4 | 3 | (information AND (interest* OR need OR needs OR question* OR asking OR ask OR seek* OR search* OR demand OR desire OR request* OR call OR requirement OR requiring OR preference*) AND (diabetes OR diabetic OR niddm OR iddm OR t2dm OR t1dm OR prediabetes OR prediabetic OR pre-diabetes OR pre-diabetic OR impaired glucose)):ti |
| #5 | 32 | ((need OR needs OR seek* OR search* OR demand OR desire OR preference* OR wish OR wishes OR provision* OR expectation*) AND (diabetes OR diabetic OR prediabetes OR prediabetic OR pre-diabetes OR pre-diabetic OR impaired glucose) AND (information OR education OR support)):ti |
| #6 | 224 | (information need AND (diabetes OR diabetic OR prediabetes OR prediabetic OR pre-diabetes OR pre-diabetic OR impaired glucose)):ti,ab,kw |
| #7 | 12 | #6 AND (information OR knowledge):ti |
| #5 | 41 | #1 OR #2 OR #3 OR #4 OR #5 OR #7  Cochrane Reviews: 1  Other Reviews: 14  Trials: 12  Methods Studies: 4  Technology Assessments: 0  Economic Evaluations: 10  Cochrane Groups: 0 |

1. *Web of Science (Thomson Reuters)*

Database: Web of Science (1950-2014)

Research period: unlimited

| **Search step** | **Hits** | **Search** |
| --- | --- | --- |
| 1 | 123 | TITLE: (information AND (interest* OR need OR needs OR question* OR talk* OR ask* OR online communi* OR seek* OR search* OR demand OR desire OR request* OR call OR requir* OR perception* OR preference*) AND (diabetes OR diabetic OR prediabetes OR prediabetic OR pre-diabetes OR pre-diabetic OR impaired glucose)) OR  TITLE: ((need OR needs OR seek* OR search* OR demand OR desire OR preference* OR wish OR wishes OR provision* OR expectation*) AND (diabetes OR diabetic OR prediabetes OR prediabetic OR pre-diabetes OR pre-diabetic OR impaired glucose) AND (information OR education OR support OR perception*)) OR  TITLE: ((education OR communication) AND (need OR needs OR preference*) AND (diabetes OR diabetic OR prediabetes OR prediabetic OR pre-diabetes OR pre-diabetic OR impaired glucose))  Timespan=All Years  Lemmatization=On |

1. *ERIC (Institute of Education Sciences)*

Database: ERIC (1966-2014)

Research period: unlimited

| **Search step** | **Hits** | **Search** |
| --- | --- | --- |
| 1 | 6 | "INFORMATION NEED*" AND "DIABETES" |

1. *ScienceDirect (Elsevier)*

Database: ScienceDirect (Elsevier, Segmente “Decision Sciences, Medicine”, “Medicine and Dentistry”, “Neuroscience”, “Nursing and Health professions”, “Pharmacology, Toxicology and Pharmaceutical Science”, “Psychology”, ”Social Sciences”) (1823-2014)

Research period: unlimited

| **Search step** | **Hits** | **Search** |
| --- | --- | --- |
| 1 | 9 | TITLE((information) AND (diabetes OR diabetic)) AND TITLE-ABSTR-KEY((interest* OR need OR needs OR question* OR ask* OR talk* OR online communi* OR seek* OR search* OR demand OR desire OR request* OR call OR requir* OR perception* OR preference*)) |

1. *CCMed, Deutsches Ärzteblatt (DIMDI)*

Database: CCMed (2001-2014), Deutsches Ärzteblatt (1996-2014)

Research period: unlimited

| **Search step** | **Hits** | **Search** |
| --- | --- | --- |
| 1 | 3 | FT=(diabetes ; diabetic ; pre-diabet? ; prediabet? ; impaired glucose ) AND TI=(information? ; education ; knowledge )) AND FT=(interest? ; need? ; question? ; talk? ; online communi? ; ask? ; seek? ; search? ; demand ; desire ; request? ; call ; requir? ; perception? ; preference?) |
| 2 | 9 | FT=(diabetes ; diabetic ; pre-diabet? ; prediabet? ; impaired glucose ) AND FT=informationsbedarf |
| 3 | 12 | 1 OR 2 |

*10. Karlsruher virtueller Katalog (KvK)(Karlsruher Institut für Technologie)*

Database: Karlsruher virtueller Katalog (DIMDI)

Research period: unlimited

| **Search step** | **Hits^1^** | **Search** |
| --- | --- | --- |
| 1 | 0 | information need* AND (diabet* OR pre-diabet* OR prediabet*) |
| 2 | 0 | informationsbedarf AND (diabet* OR pre-diabet* OR prediabet*) |
| 3 | 0 | preferences AND (diabet* OR pre-diabet* OR prediabet*) |

^1^after Screening
